# Effects of dietary macronutrients on the hepatic transcriptome and serum metabolome in mice

**DOI:** 10.1111/acel.13585

**Published:** 2022-03-10

**Authors:** Yingga Wu, Cara L. Green, Guanlin Wang, Dengbao Yang, Li Li, Baoguo Li, Lu Wang, Min Li, Jianbo Li, Yanchao Xu, Xueying Zhang, Chaoqun Niu, Sumei Hu, Jacques Togo, Mohsen Mazidi, Davina Derous, Alex Douglas, John R. Speakman

**Affiliations:** ^1^ State Key Laboratory of Molecular Developmental Biology Institute of Genetics and Developmental Biology Chinese Academy of Sciences Beijing People’s Republic of China; ^2^ University of Chinese Academy of Sciences Beijing People’s Republic of China; ^3^ Institute of Biological and Environmental Sciences University of Aberdeen Aberdeen Scotland UK; ^4^ Shenzhen Key Laboratory of Metabolic Health Center for Energy Metabolism and Reproduction Shenzhen Institutes of Advanced Technology Chinese Academy of Sciences Shenzhen People’s Republic of China; ^5^ University of Dali Dali Yunnan Province People’s Republic of China; ^6^ CAS Center of Excellence in Animal Evolution and Genetics Kunming People’s Republic of China

**Keywords:** aging, carbohydrate, fat, metabolome, protein, transcriptome

## Abstract

Dietary macronutrient composition influences both hepatic function and aging. Previous work suggested that longevity and hepatic gene expression levels were highly responsive to dietary protein, but almost unaffected by other macronutrients. In contrast, we found expression of 4005, 4232, and 4292 genes in the livers of mice were significantly associated with changes in dietary protein (5%–30%), fat (20%–60%), and carbohydrate (10%–75%), respectively. More genes in aging‐related pathways (notably mTOR, IGF‐1, and NF‐kappaB) had significant correlations with dietary fat intake than protein and carbohydrate intake, and the pattern of gene expression changes in relation to dietary fat intake was in the opposite direction to the effect of graded levels of caloric restriction consistent with dietary fat having a negative impact on aging. We found 732, 808, and 995 serum metabolites were significantly correlated with dietary protein (5%–30%), fat (8.3%–80%), and carbohydrate (10%–80%) contents, respectively. Metabolomics pathway analysis revealed sphingosine‐1‐phosphate signaling was the significantly affected pathway by dietary fat content which has also been identified as significant changed metabolic pathway in the previous caloric restriction study. Our results suggest dietary fat has major impact on aging‐related gene and metabolic pathways compared with other macronutrients.

## Graphical abstract

More hepatic genes in aging‐related gene pathways mTOR, IGF1, and NF‐kappaB had significant correlations with dietary fat intake than protein and carbohydrate intakes. Using serum metabolomics, the sphingosine‐1‐phosphate signaling which has been identified as aging‐related metabolic pathway was significantly affected only by dietary fat content. Therefore, the dietary fat content had significant impact on markers of aging in mice.

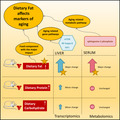

## 1 INTRODUCTION

The food we eat consists of three macronutrients: protein, fat, and carbohydrates. Understanding the different impacts of these macronutrients on health, metabolism, and aging are key goals. Absorbed food is first processed by the liver, which is accordingly an important regulator of several metabolic processes including lipid, glucose, and amino acid metabolism. Liver lipid content can be affected by dietary macronutrient composition via effects on hepatic lipogenesis, fatty acid oxidation, and triglyceride synthesis (Postic & Girard, 2008; de Wit et al., 2012). Diets high in fat result in increased hepatic lipids and may result in fatty liver disease in both rodents and humans (Patsouris et al., 2006; Westerbacka et al., 2005). In contrast, high protein diets have been suggested to prevent hepatic lipid accumulation (Lacroix et al., 2004; Shertzer et al., 2011). Despite these potentially protective effects against hepatic steatosis, several studies have suggested that high protein diets are linked to increases in all caused mortality (Díaz‐Rúa et al., 2017). The negative effects of high protein diets have been indicated to be potentially associated with metabolites produced in different stages of amino acid metabolism (Díaz‐Rúa et al., 2017). Moreover, increased dietary protein to carbohydrate ratios lead to upregulation of genes involved in amino acid uptake and fatty acid synthesis, reflecting increased triacylglycerol content and increased health risk (Díaz‐Rúa et al., 2017). In contrast, several studies which explored amino acid restriction or protein restriction effects on metabolism of mice, including, for example, leucine deprivation and methionine restriction, showed downregulation of liver lipogenic genes (Anthony et al., 2013; Guo & Cavener, 2007; Laeger et al., 2014).

Several recent studies indicated that fibroblast growth factor 21 (FGF21) was upregulated when feeding on a low protein diet. This led to speculation that FGF21 is an endocrine signal of protein restriction and the beneficial effects of protein restriction on metabolic health might be dependent on FGF21 (Laeger et al., 2014, 2016). Apart from comparisons of high versus low protein diets, recent work has investigated gene expression levels in mice fed a matrix of diets varying in their protein, carbohydrate, and fat content (Gokarn et al., 2018). This revealed that dietary protein intake had a powerful effect on hepatic gene expression compared with dietary carbohydrate and fat content, and also showed that dietary protein mostly affected mitochondrial function and amino acid metabolism pathways, simultaneous to upregulation of *Fgf21* at lower protein intakes (Gokarn et al., 2018). In contrast, the impacts of dietary fat and carbohydrate on hepatic gene expression were negligible (Gokarn et al., 2018).

Dietary macronutrient composition also has profound effects on aging (Gokarn et al., 2018). Several studies indicated that low protein high carbohydrate diets increase rodent lifespan (Lee et al., 2008). Consistent with these lifespan impacts of lowered protein, in the most comprehensive long‐term study of different dietary macronutrient composition effects on liver gene expression patterns, it was found that dietary protein intake affected several major nutrient sensing pathways linked to aging, including adenosine‐5‐monophosphate‐activated protein kinase (AMPK), mammalian target of rapamycin (mTOR), insulin‐like growth factor (IGF‐1), and FGF21 signaling (Gokarn et al., 2018). The liver shows relatively fewer significant changes with age compared to other organs (Schmucker & Sanchez, 2011); however, the number of mitochondria per hepatocyte decreases with age in both rodents and humans (de la Cruz et al., 1990; Herbener, 1976). A study of mitochondrial dysfunction in the obese rat suggested that mitochondrial changes consequent of fat intake may be the cause of aging and age‐related disorders in obesity (Rector et al., 2010).

Apart from protein restriction, the most frequently studied nutritional impact on aging is caloric restriction. It has been shown that caloric restriction increases lifespan and decreases age‐related diseases in many species (Ingram & de Cabo, 2017; Mercken et al., 2012). In mice and other organisms, several nutrient sensing pathways have been implicated to mediate the beneficial caloric restriction effect on aging. These pathways include decreased IGF‐1 signaling (Argentino et al., 2005; Breese et al., 1991), reduced mTOR signaling (Johnson et al., 2013), and reduced nuclear factor‐kappa beta (NF‐kB) signaling (Tilstra et al., 2011). All three pathways were modified in relation to the level of restriction in a way that indicated improved aging in the livers of mice (Derous et al., 2017). Caloric restriction protocols however often reduce all the macronutrients at the same time. Hence the contribution of specific macronutrient reductions to these changes is not clear. It has been suggested that much of the effect of CR might be mediated not by lowered calorie intake but by lowered protein intake (Solon‐Biet et al., 2014, 2019), but this claim is disputed (Speakman et al., 2016).

In the present study, we investigated the effects on mouse hepatic gene expression (by RNA‐seq) of ad libitum intake of six different levels of dietary protein (5%–30%) combined with both high‐fat (60%) and low‐fat (20%) conditions, leading to 12 different levels of dietary carbohydrate (10%–75%). In addition, we explored the impacts of 24 different diets (varying from 5% to 30% protein, 8.3% to 80% fat, and 10% to 80% carbohydrate) on serum metabolite levels by untargeted metabolomics.

## 2 RESULTS

### 2.1 Impact of dietary macronutrient composition on hepatic gene expression

**FIGURE 1:**
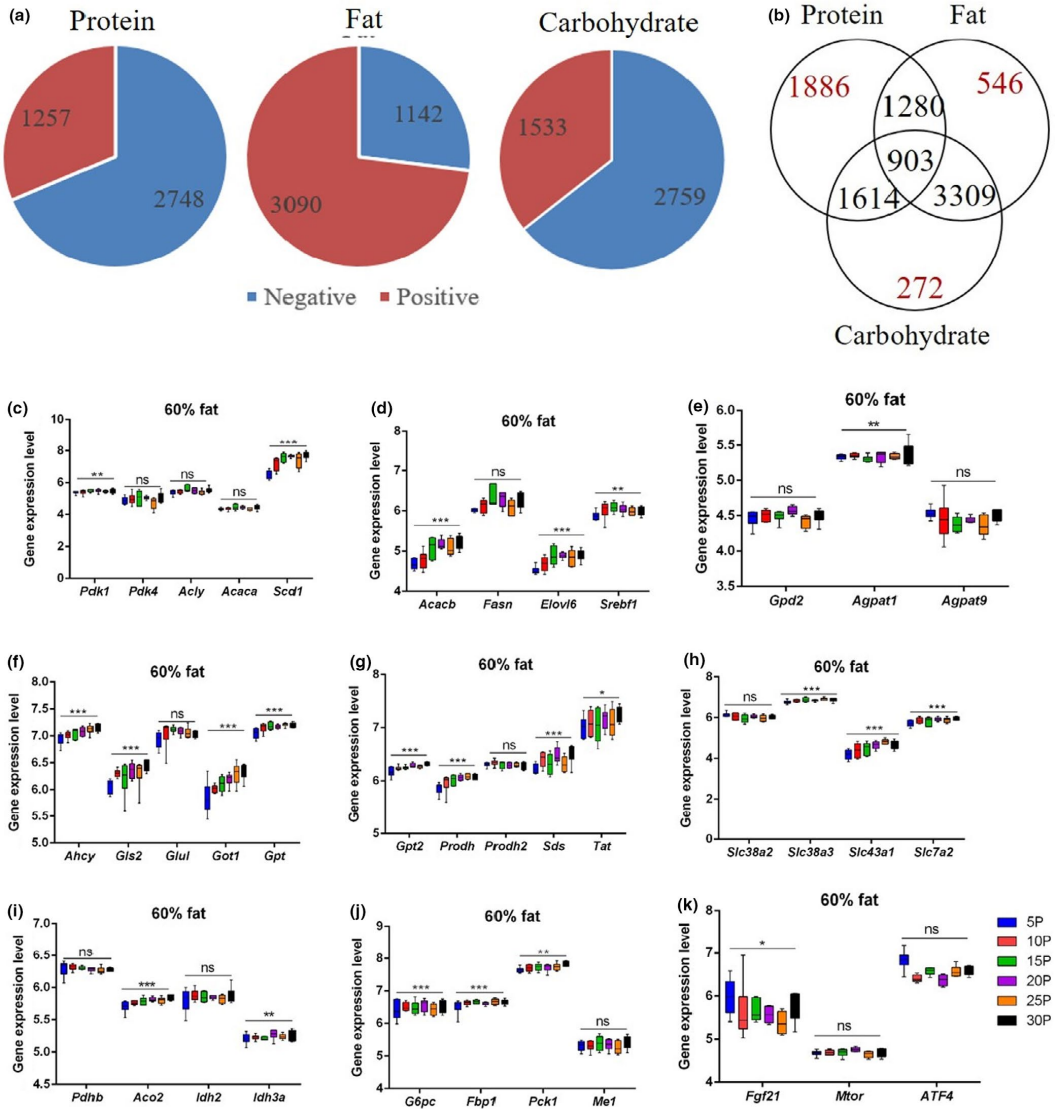
Diagram showing genes correlated with dietary protein, fat, and carbohydrate contents and gene expression patterns in several metabolic pathways. (a) The total number of genes significantly correlated, respectively, with dietary protein, fat, and carbohydrate contents. (b) Overlapped and independent correlated genes with dietary protein, fat, and carbohydrate contents. (c and d) Fatty acid synthesis metabolism, (e) triglyceride synthesis metabolism, (f, g) amino acid metabolism, (h) amino acid transport metabolism, (i) TCA cycle, (j) gluconeogenesis metabolism, and (k) regulation of protein intake‐related genes (n = 5–6). Generalized linear modeling was performed to analyze the dietary protein effect on specific gene expression. * *p* < 0.05, ** *p* < 0.01, *** *p* < 0.001, ns *p* > 0.05. Values are represented as mean ±SD

Pearson correlation analysis indicated that expression of 4005 genes was significantly correlated with dietary protein content (2748 negative correlation and 1257 positive correlation) and 4232 genes (1142 negative correlation and 3090 positive correlation) were significantly associated with dietary fat content, whereas expression of 4292 genes (2759 negative correlation 1533 positive correlation) was associated with dietary carbohydrate levels (Figure 1a). We analyzed data on hepatic gene expression using generalized linear modeling (GLM) with gene expression of each gene as the dependent variable and dietary levels of fat, protein, and carbohydrate content, and the interactions of the macronutrients as the independent predictors. We found five key genes involved in fatty acid synthesis pyruvate dehydrogenase kinase 1 (*Pdk1*), stearoyl‐CoA desaturase 1 (*Scd1)*, acetyl‐CoA carboxylase beta (*Acacb*), ELOVL family member 6 (*Elovl6*), and Sterol regulatory element‐binding transcription factor 1 (*Srebf1*) were all significantly upregulated in relation to the increase of dietary protein (*p* = 0.005, *p* = 2.4 × 10^−5^, *p* = 2.3 × 10^−5^, *p* = 0.006, *p* = 0.002, respectively), but for other genes involved in fatty acid synthesis pyruvate dehydrogenase kinase 4 (*Pdk4*), ATP citrate lyase (*Acly*), acetyl‐CoA carboxylase alpha (*Acaca*), fatty acid synthase (*Fasn*), there were no significant associations with dietary protein contents (*p* > 0.05) (Figure 1c, d and Figure S1a, b). The triglyceride synthesis‐related gene 1‐acylglycerol‐3‐phosphate O‐acyltransferase 1 (*Agpat1*) was also significantly positively associated with the dietary protein (*p* = 0.002), but glycerol‐3‐phosphate dehydrogenase 2 (*Gpd2*) and 1‐acylglycerol‐3‐phosphate O‐acyltransferase 9 (*Agpat9*) were not significantly associated (Figure 1e and Figure S1c).

Expression of many genes related to amino acid metabolism was also strongly altered in relation to dietary protein levels, for example, adenosylhomocysteinase (*Ahcy*), glutaminase 2 (*Gls2*), glutamic‐oxaloacetic transaminase (*Got1*), glutamic‐pyruvic transaminase (*Gpt*), glutamic‐pyruvic transaminase 2 (*GPt2*), proline dehydrogenase (*Prodh*), serine dehydratase (*Sds*), and tyrosine aminotransferase (*Tat*), was all significantly upregulated with increases in dietary protein content (Figure 1f,g and Figure S1d,e, *p* values in Table S10). Further amino acid transport genes solute carrier family (Slc) 38 member 3 (*Slc38a3*), *Slc43a1* and *Slc7a2* were also strongly positively related to the dietary protein contents, but not *Slc38a2* (Figure 1h and Figure S1f, *p* values in Table S10). Gene expression levels in TCA cycle genes aconitase 2 (*Aco2*) (*p* = 1.82 × 10^−8^) and isocitrate dehydrogenase 3 alpha (*Idh3a*) (*p* = 0.001) were both positively associated with the dietary protein content (Figure 1i and Figure S1g). Moreover, genes involved in gluconeogenesis such as glucose‐6‐phosphatase (*G6pc*) (*p* = 0.04), phosphoenolpyruvate carboxykinase 1 (*Pck1*) (*p* = 0.005), and fructose‐bisphosphatase 1 (*Fbp1*) (*p* = 0.007) were all significantly increased as dietary protein increased (Figure 1j and Figure S1h). Apart from these changes in the fatty acid and amino acid metabolism pathways, *Fgf21* was significantly decreased with increased dietary protein (*p* = 0.001) (Figure 1k and Figure S1i). Surprisingly, no significant correlations were observed between dietary protein content and other protein “sensing” genes such as *Mtor* and activating transcription factor 4 (*Atf4)* (*p* > 0.05) (Figure 1k and Figure S1i).

We explored the relationships between gene expression at different protein levels and body fat content and serum hormone levels. The fatty acid metabolism genes *Scd1* and *Elovl6* were significantly positively correlated with the total body fat, and with serum leptin and insulin levels (Figure S2a–S2f). The amino acid metabolism gene *Gpt* was also significantly positively related to the body fat content, serum leptin, and insulin concentration (Figure S2g–S2i). *Fgf21* in contrast was not correlated with body fat or serum hormone concentrations (Figure S2j–S2l).

**FIGURE 2:**
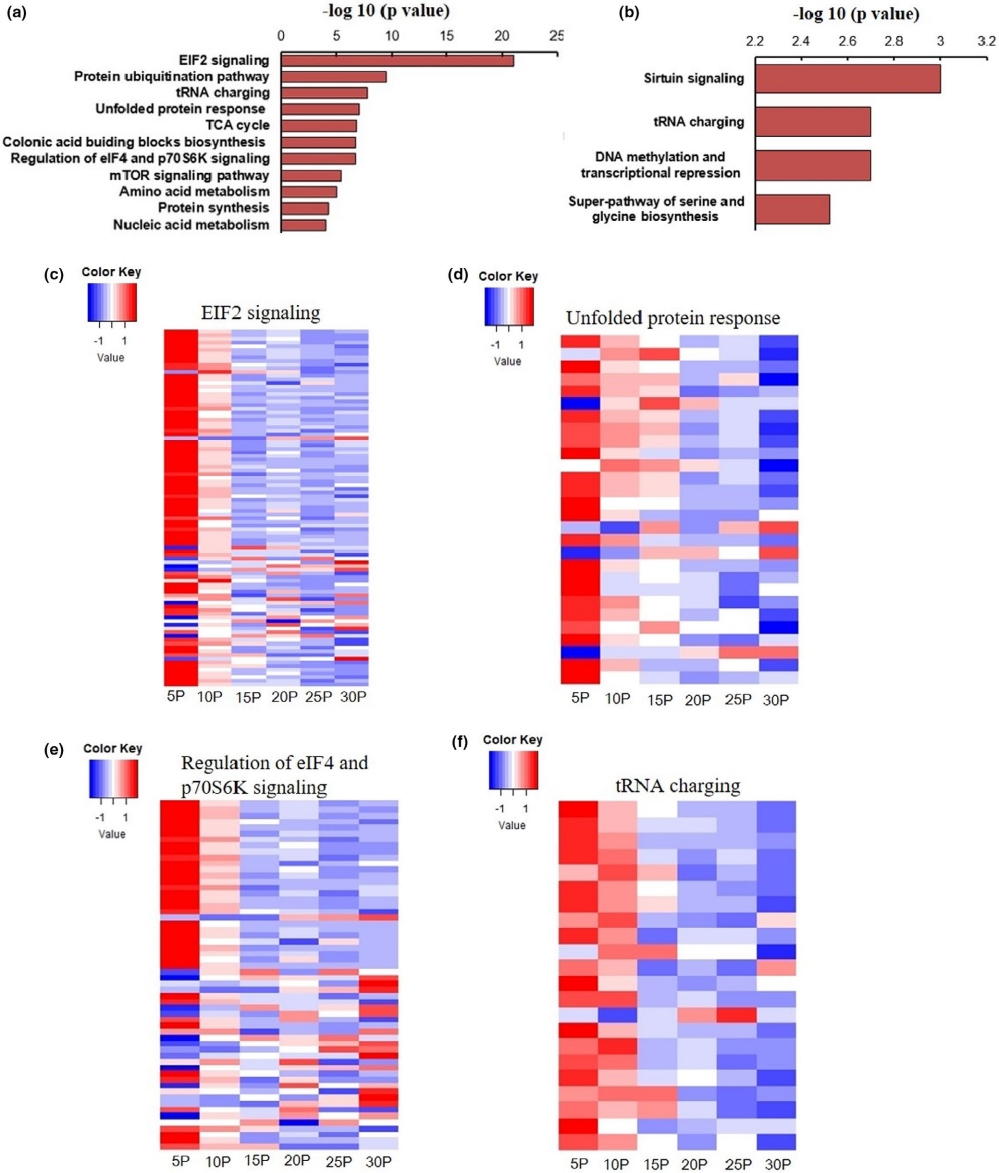
Significantly changed gene pathways with the increase of dietary protein content. (a) Significantly changed pathways related to the increasing dietary protein content. (b) Significantly changed pathways correlated independently with the increasing protein level. Gene expression patterns in different protein content groups in (c) EIF2a signaling pathway, (d) unfolded protein response, (e) regulation of eIF4 and p70S6K signaling, (f) tRNA charging pathway, blue indicates lower and red indicates higher expression. Generalized linear modeling and Pearson correlation analysis were performed to analyze the dietary protein effect on gene expression patterns

GLM analysis of liver gene expression changes indicated eukaryotic translation initiation factor 2 (EIF2) signaling (*p* = 8.11 × 10^−22^), the unfolded protein response (*p* = 8.47 × 10^−8^), regulation of eIF4 and ribosomal protein S6 kinase (p70S6K) signaling (*p* = 1.91 × 10^−7^), transfer RNA (tRNA) charging (*p* = 1.64 × 10^−8^), amino acid metabolism (*p* = 8.99 × 10^−6^), protein synthesis (*p* = 5.58 × 10^−5^), and nucleic acid metabolism pathways (*p* = 9.4 × 10^−5^) were the most significantly affected pathways with the increasing of dietary protein contents under both 60% fat and 20% fat conditions (Figure 2a,c,d,e,f) (Table S1).

There were 99/205 significant changes in gene expression in the EIF2 signaling pathway (Figure S4a), most of these genes (94/99) were negatively correlated with the dietary protein content. In the regulation of eIF4 and p70S6K signaling pathway, 57/157 genes showed significant expression changes with the elevation of dietary protein contents and also most of the altered genes (50/57 genes) were downregulated (Figure S3a). The tRNA charging pathway in the liver was also significantly affected by dietary protein content, including 23/38 genes that were significantly changed in this pathway, and all of which (23/23) were negatively correlated with the protein content in the diet. Changes of unfolded protein response pathway also reflect the end of the protein translation process, and there were 28/55 genes significantly changed in this pathway, of which 26/28 genes had negative correlations with dietary protein contents (Figure S3b). Pearson correlation results showed that 4005 genes were significantly correlated with protein level in the diet, and the following pathway analysis results revealed that EIF2a signaling (*p* = 5.09 × 10^−17^), TCA cycle (*p* = 1.48 × 10^−7^), the unfolded protein response (*p* = 2.13 × 10^−7^), regulation of eIF4 and p70S6K signaling (*p* = 6.82 × 10^−8^), protein ubiquitination pathway (*p* = 3.29 × 10^−10^), tRNA charging (*p* = 3.33 × 10^−8^), colonic acid building blocks biosynthesis pathway (*p* = 1.68 × 10^−7^), and the mTOR signaling pathway (*p* = 3.53 × 10^−6^) were the most significantly changed pathways (Figure 2a) (Table S1). Of the 4005 genes, only 1886 genes were independently correlated with protein content in the diet (1614 genes were correlated both with protein and carbohydrate levels and 1280 genes were associated with protein and fat content in the diet) (Figure 1b). IPA pathway analysis of these 1886 genes showed that sirtuin signaling (*p* = 0.001), tRNA charging (*p* = 0.002), DNA methylation and transcriptional repression signaling (*p* = 0.002), and the super‐pathway of serine and glycine biosynthesis (*p* = 0.003) were the most significantly affected pathways independently related to dietary protein content (Figure 2b) (Table S1).

**FIGURE 3:**
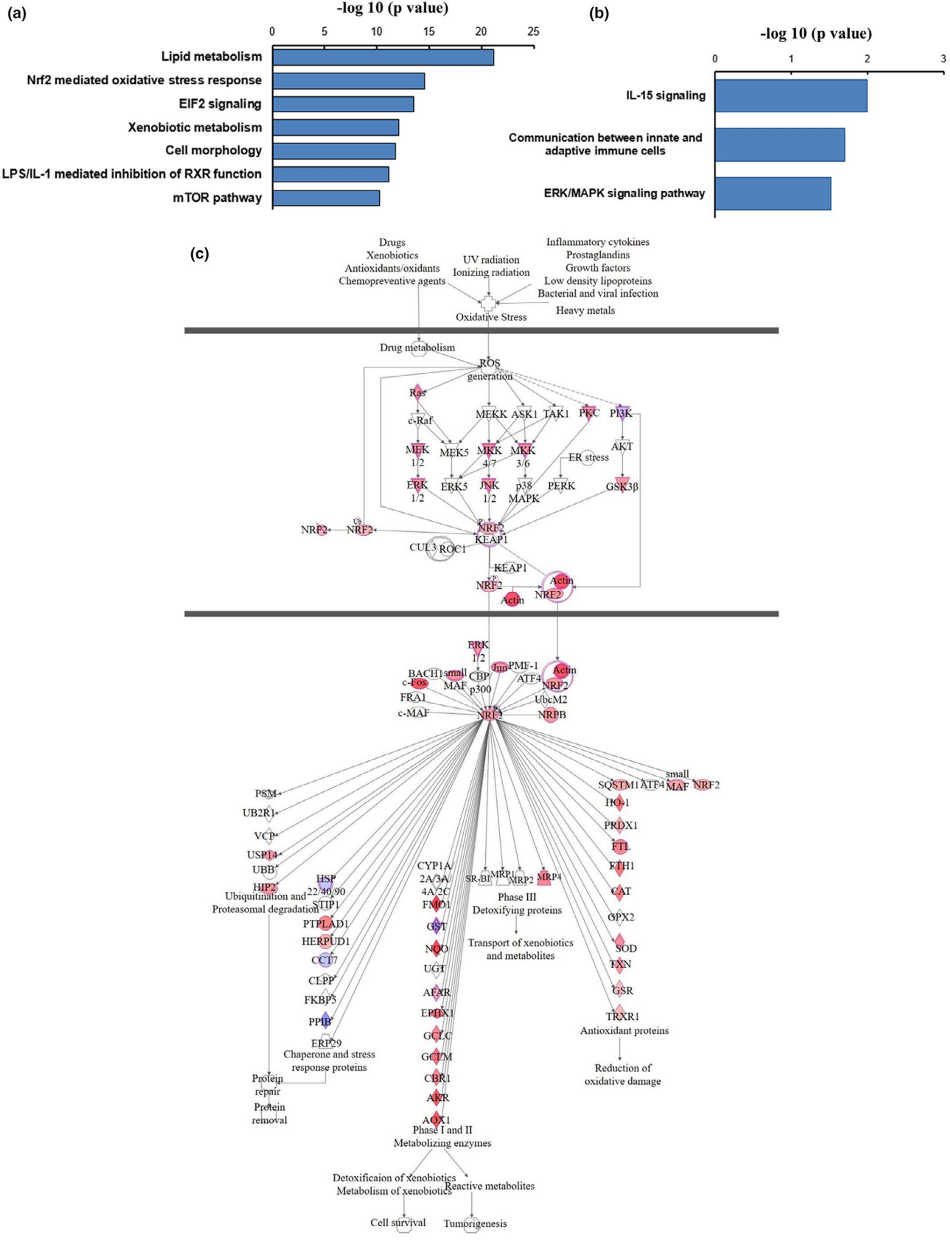
Significantly changed gene pathways with the increase of dietary fat content. (a) Significantly changed gene pathways related to the increasing dietary fat content. (b) Significantly changed gene pathways correlated independently with the increasing fat level. (c) Nrf2‐mediated oxidative stress response, red indicates positive and blue indicates the negative regression with the fat content in the diet, gray indicates no significance. Pearson correlation was performed to analyze the dietary fat effect on gene expression patterns

There were 4232 genes significantly correlated with the increasing dietary fat level (3309 genes were correlated both with fat and carbohydrate levels and 1280 genes were associated with protein and fat content in the diet, 546 genes were independently related to the dietary fat content) (Figure 1a, b). Liver gene expression changes with different fat content diets (using all 4232 genes correlated with dietary fat) indicated the lipid metabolism (*p* = 6.32 × 10^−22^), nuclear factor, erythroid 2 like 2 (Nrf2)‐mediated oxidative stress response pathway (*p* = 2.83 × 10^−15^), EIF2a signaling (*p* = 3.24 × 10^−14^), lipopolysaccharide/interleukin‐1 (LPS/IL‐1)‐mediated inhibition of retinoid X receptors (RXR) function (*p* = 7.5 × 10^−12^), xenobiotic metabolism (*p* = 7.59 × 10^−13^), cell morphology (*p* = 1.74 × 10^−12^), and the mTOR signaling pathway (*p* = 5.62 × 10^−11^) were the most significantly changed pathways (Figure 3a). There were 82/179 significant changes in gene expression in the Nrf2‐mediated oxidative stress response pathway (Figure 3), most of these genes (74/82) were positively correlated with the dietary fat contents (Figure 3c) (Table S2). In the LPS/IL‐1 mediated inhibition of RXR function and mTOR signaling pathway, 83/205 and 79/198 genes showed significant expression changes with the elevation of dietary fat contents, respectively (Figure S4b). Furthermore, the pathway analysis of the independent significantly changed genes (546 genes) with the increasing of dietary fat content indicated that IL‐15 signaling (*p* = 0.01), communication between innate and adaptive immune cells (*p* = 0.02), ERK/MAPK signaling pathway (*p* = 0.03) were the significantly affected pathways separately correlated with the fat content in the diet (Figure 3b) (Table S2).

There were 4292 genes significantly correlated with increasing dietary carbohydrate content, and following pathway analysis results showed that EIF2 signaling (*p* = 4.14 × 10^−23^), xenobiotic metabolism signaling (*p* = 3.52 × 10^−13^), LPS/IL‐1 mediated inhibition of RXR function (*p* = 1.08 × 10^−12^), NRF‐2‐mediated oxidative stress response (*p* = 2.97 × 10^−10^), and mTOR signaling pathway (*p* = 3.18 × 10^−9^) were the most significantly changed pathways (Figure S5a,c and Table S3). These pathways overlapped with pathways affected by dietary protein and fat content because most of these 4292 genes were also correlated both with dietary protein (1614 genes) and fat (3309 genes) contents (Figure 1a, b). Therefore, we selected genes separately correlated with carbohydrate content (272 genes) and performed pathway analysis on these genes. We found methionine degradation (*p* = 0.001), cysteine biosynthesis (*p* = 0.002), BEX2 signaling (*p* = 0.003), and Wnt/‐catenin signaling (*p* = 0.005) were the most significantly changed pathways with increasing carbohydrate content (Figure S5b and Table S3).

**FIGURE 4:**
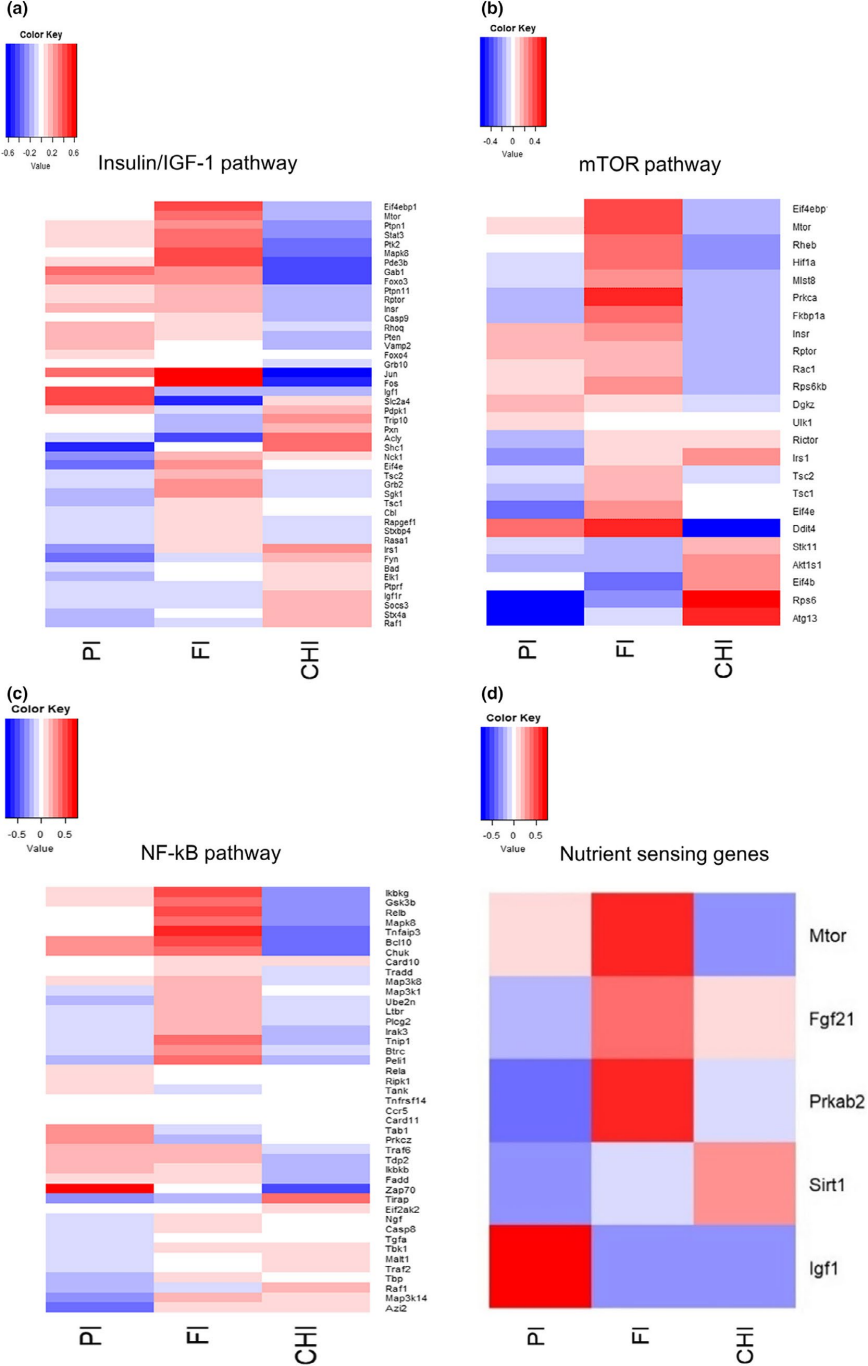
Relationship between aging‐related genes and macronutrient intakes. (a‐c) The relationship between gene expression levels in aging pathways (a) insulin/IGF‐1 pathway, (b) mTOR pathway, (c) NF‐kB pathway and protein intake (PI), fat intake (FI), and carbohydrate intake (CHI). (d) The correlations between macronutrient intakes (PI, FI, CHI) and nutrient sensing genes. Pearson correlation method was used for statistical analysis, the color key is the correlation coefficients of Pearson correlation analysis between gene expression levels and macronutrient intakes

Insulin/IGF‐1, mTOR, and NF‐kB signaling are three important pathways involved in aging (Derous et al., 2017). In the insulin/IGF‐1 signaling pathway, there were 4/46 genes significantly positively and also 4/46 genes negatively correlated with protein intake, 2/46 genes were significantly negatively and 13/46 genes were positively associated with the fat intake, whereas 8/46 genes were negatively and 3/46 genes positively correlated with the carbohydrate intake (Figure 4a) (Table S4). *Igf1* was only significantly positively correlated with protein intake (*p* = 0.0003, r = 0.427) with no significant correlations with carbohydrate and fat intakes (Table S4). In the mTOR signaling pathway, there were more genes correlated negatively with protein intake (4/24 negative associated genes and 1/24 positive associated gene), whereas 2/24 genes had significant negative and 8/24 genes had positive correlation with fat intake (Figure 4b). Also relatively few genes in this pathway had a negative correlation with the carbohydrate intake (1/24 negative associated genes and 3/24 positive associated gene) (Figure 4b) (Table S5). Expression of *Mtor* itself was only significantly positively associated with the dietary fat intake (*p* = 0.003, r = 0.357) (Table S5). In the NF‐kB signaling pathway, almost half of the genes that changed had positive and another half had negative relationships with dietary protein intake (3/43 negative correlated genes and 5/43 positive correlated genes), whereas slightly more genes correlated with fat intake (0/43 negative correlated genes and 10/43 positive correlated genes) and carbohydrate intake (9/43 negative correlated genes and 1/43 positive correlated genes) (Figure 4c) (Table S6). Overall, there were almost the same number of genes had both positive and negative correlations with dietary protein intakes in IGF‐1 and NF‐kB pathway. Expression of more genes was negatively correlated in the mTOR pathway with dietary protein intakes. In contrast, more genes had negative relationships with carbohydrate intake in both IGF‐1 and NF‐kB signaling pathway, whereas in all three aging pathways, there were many more genes that had positive associations with dietary fat intake. These latter changes were in the opposite direction to the previously established effect of graded levels of caloric restriction on the same pathways (Derous et al., 2017). Apart from these aging gene expression patterns, in five nutrient sensing genes *Mtor*, *Fgf21*, *Igf1*, sirtuin 1 (*Sitr1*), protein kinase AMP‐activated non‐catalytic subunit beta 2 (*Prkab2*) that were indicated to link nutrient intake with aging in previous studies, only *Igf1* (*p* = 0.0003, r = 0.427) and *Prkab2* (*p* = 0.036, r = −0.254) were significantly associated with dietary protein intake and no genes were significantly associated with carbohydrate intake, whereas *Mtor* (*p* = 0.003, r = 0.357) and *Prkab2* (*p* = 0.003, r = 0.358) had significant positive correlations with fat intake (Figure 4d).

We also performed Pearson correlation analysis between liver weight, hormone levels, and dietary macronutrient composition. There was no significant correlation between liver weight (both wet and dry weight) and dietary protein content, whereas dry liver weight had significant negative correlation with dietary fat content (*p* = 1 × 10^−4^, r = −0.44) (Figures S6a‐c). The liver triglyceride concentration had significant positive correlation with dietary fat level (*p* = 0.03, r = 0.47), but had no significant relationship with dietary protein content (Figures S6d‐f). In contrast, the glycogen concentration was significantly correlated with dietary protein content in the lowfat group (*p* = 0.003, r = −0.62). Dietary fat content had no significant impact on glycogen concentration (Figures S6g‐i). Among genes (Scd1, Srebf1, Mttp, Ppara, Eif4ebp1, Acsl1, Gck, Tat, Pck1, Angptl4) have been indicated to be involved in the regulation of triglyceride (Li et al., 2020; Nishikawa et al., 2012; Toyoshima et al., 2020) and glycogen concentration (Præstholm et al., 2021; Ruiz et al., 2014), Acsl1 and Gck (*p* = 0.038, r = 0.32 and *p* = 0.001, r = −0.48) had significant correlations with triglyceride and glycogen concentrations respectively.

### 2.2 Impact of dietary macronutrient composition on transcription factors

To identify the key transcription factors that were over or underrepresented in terms of their binding sites in regulatory regions of significantly correlated genes with dietary protein, fat, or carbohydrate when compared to background genes (had no significant correlations), we performed enrichment analysis by using the CiiiDER software. The analysis revealed that nuclear factor I X (NFIX) and distal‐less homeobox 2 (Dlx2) were mostly enriched in the promoters of genes significantly correlated both with dietary protein and carbohydrate (*p* = 5.51 × 10^−12^, *p* = 6.25 × 10^−11^ for protein and *p* = 5.34 × 10^−14^, *p* = 4.56 × 10^−7^ for carbohydrate), whereas E74 like ETS transcription factor 4 (ELF4) and Dlx2 were recognized as most enriched transcription factors of genes that had significant associations with dietary fat content (*p* = 1.19 × 10^−11^, *p* = 6.78 × 10^−11^) (Figure S6j. k). We also performed Pearson correlation analysis between significantly enriched transcription factors and gene expression levels in significantly affected gene pathways by different dietary macronutrients. There were 17/99 genes significantly correlated with NFIX in the EIF2a signaling pathway (significantly affected pathway by dietary protein content). 14/82 genes had significant correlations with ELF4 transcription factor in the Nfr2 signaling pathway (significantly affected pathway by dietary fat content). The key transcription factors peroxisome proliferator‐activated receptors (PPARs) (PPARα, PPARγ, PPARδ in this study) that have been shown to respond to dietary macronutrients (Kersten et al., 1999) were only significantly enriched in the promoter regions of genes correlated with dietary fat content but not with dietary protein or carbohydrate content.

### 2.3 Impact of dietary macronutrient composition on circulating metabolites

**FIGURE 5:**
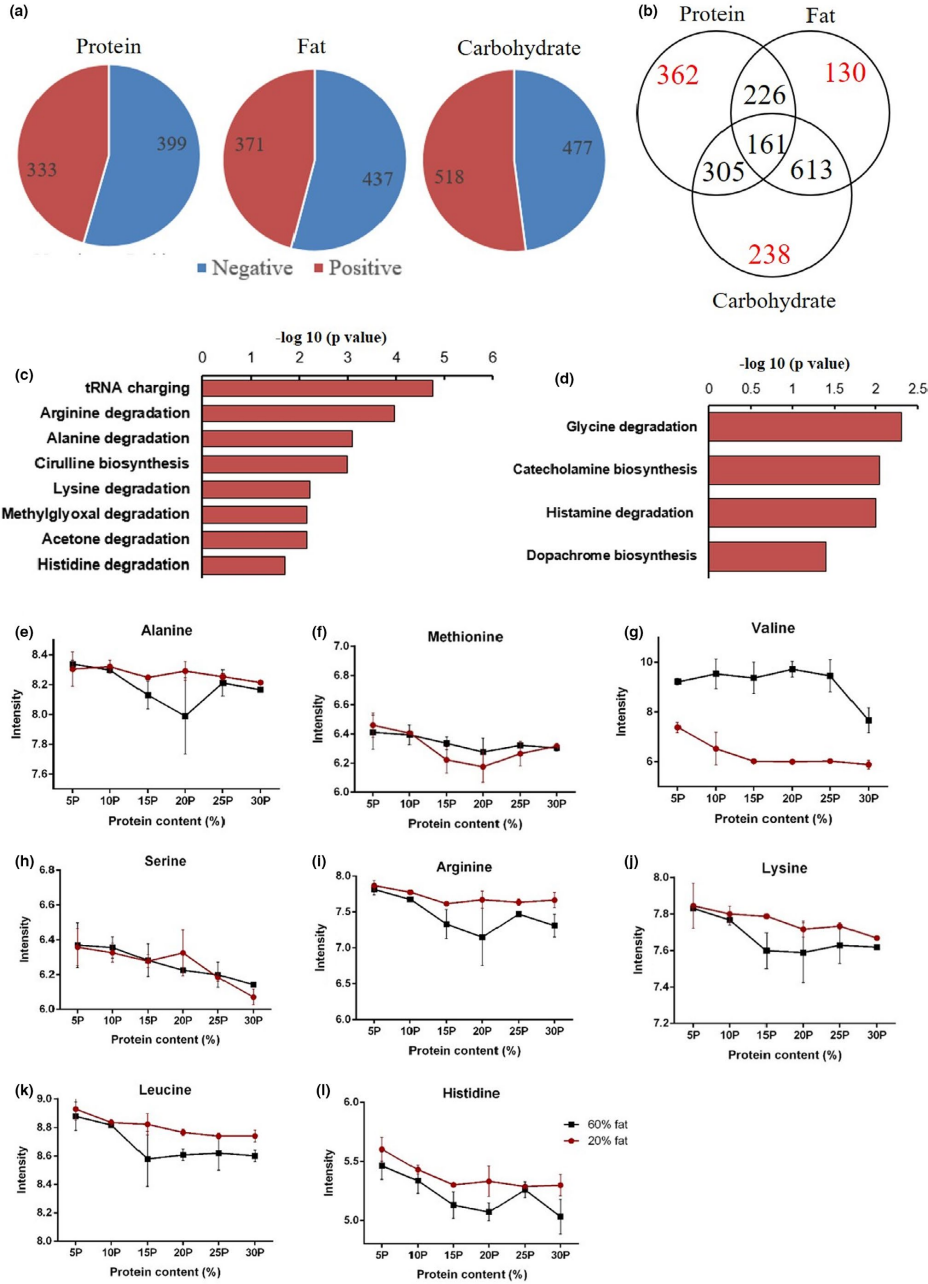
Diagram showing metabolites correlated with dietary protein, fat, and carbohydrate contents and significantly changed metabolic pathways and metabolites in the serum of mice fed different protein content diets. (a) The total number of metabolites significantly correlated, respectively, with dietary protein, fat, and carbohydrate contents. (b) Overlapped and independent correlated metabolites with dietary protein, fat, and carbohydrate contents. (c) Significantly changed metabolic pathways related to the increasing dietary protein content. (d) Significantly changed metabolic pathways correlated independently with the increasing protein level. (e‐l) Log‐transformed concentration of alanine, methionine, valine, serine, arginine, lysine, leucine, and histidine in different protein content diet treatment groups, respectively. Generalized linear modeling and Pearson correlation were performed to analyze the dietary protein effect on gene expression patterns

Circulating levels of 732 metabolites were significantly correlated with dietary protein content (399 negative and 333 positive correlations), 808 metabolites (437 negative and 371 positive correlations) were significantly correlated with dietary fat content, and 995 metabolites (477 negative and 518 positive correlations) were correlated with dietary carbohydrate levels (Figure 5a).

We performed GLM analysis for amino acids with dietary protein content as a covariate and fat content of the diet (2 levels) as a factor. Serum alanine (*p* = 0.003), methionine (*p* = 0.013), valine (*p* = 0.019), serine (*p* = 0.023), arginine (*p* < 0.001), lysine (*p* = 1.95 × 10^−5^), leucine (*p* < 0.001), and histidine (*p* = 7.68 × 10^−5^) amino acid levels were all significantly negatively correlated with dietary protein content (Figure 5e‐5l). However, the concentrations of other amino acids (phenylalanine, proline, aspartic acid, cysteine, glycine, tyrosine, tryptophan, glutamate, and threonine) were not significantly associated with the dietary protein levels (Figure S7b, c). To further explore correlations between significantly changed metabolites and some important physiological traits, we also correlated the metabolites (Pearson's correlation) with body fat, serum hormones, and macronutrient intakes. We found all the amino acids that decreased significantly with protein content were significantly negatively correlated with the body fat levels and serum leptin concentrations (Figure S7a) (p and r values in Table S10). Several of them (lysine, arginine, histidine, and leucine) had strong associations with the serum insulin concentration (Figure S7a) (p and r values in Table S10).

In addition to looking at the relationships between metabolites and the dietary macronutrient contents (above), we also sought relationships between them and actual intakes. Alanine, methionine, valine, serine, arginine, lysine, leucine, and histidine were all significantly decreased with increasing dietary protein intake, whereas they increased with the elevation of dietary carbohydrate intakes (Figure S7a) (p and r values in Table S10). As for the correlation with fat intake, only arginine concentration was significantly related to the dietary fat intake (*p* = 0.02) (Figure S7a).

To explore the significantly affected metabolite pathways we also performed the GLM analysis for all metabolites and then selected metabolites significantly correlated with dietary protein content (*p* < 0.05) for upload into the Ingenuity pathway analysis (IPA) software. The most affected pathways included tRNA charging (*p* = 1.7 × 10^−5^), arginine degradation (*p* = 1.1 × 10^−4^), alanine degradation (*p* = 8.05 × 10^−4^), and citrulline biosynthesis (*p* = 0.001) (Figure 5c) (Table S7). Pearson correlation analysis with dietary protein levels across all metabolites (732 metabolites), respectively, in the 60% fat group and 20% fat group also indicated the most affected pathways were tRNA charging (*p* = 0.001 for high‐fat group, *p* = 1.71 × 10^−6^ for low‐fat group), arginine degradation (*p* = 3.45 × 10^−5^), alanine degradation (*p* = 4.49 × 10^−4^), and citrulline biosynthesis (*p* = 4.44 × 10^−4^) (Figure 5c). Additionally, lysine degradation (*p* = 0.006), methylglyoxal degradation (*p* = 0.007), acetone degradation (*p* = 0.007), and histidine degradation (*p* = 0.02) pathways were also recognized as significantly changed pathways (Figure 5c) (Table S7). Furthermore, in 732 metabolites, 305 metabolites were correlated both with dietary protein and carbohydrate content and 226 metabolites were associated both with protein and fat content in the diet (Figure 5b). There were 362 metabolites related only with the dietary protein content, and the following pathway analysis of these 362 metabolites indicated that glycine degradation (*p* = 0.005), catecholamine biosynthesis (*p* = 0.009), histamine degradation (*p* = 0.01), and dopachrome biosynthesis (*p* = 0.04) were significantly changed pathways with increasing dietary protein content (Figure 5d) (Table S7). Overall, the most significantly changed metabolites and metabolic pathways related to increasing dietary protein levels were mainly related to the amino acid metabolism.

**FIGURE 6:**
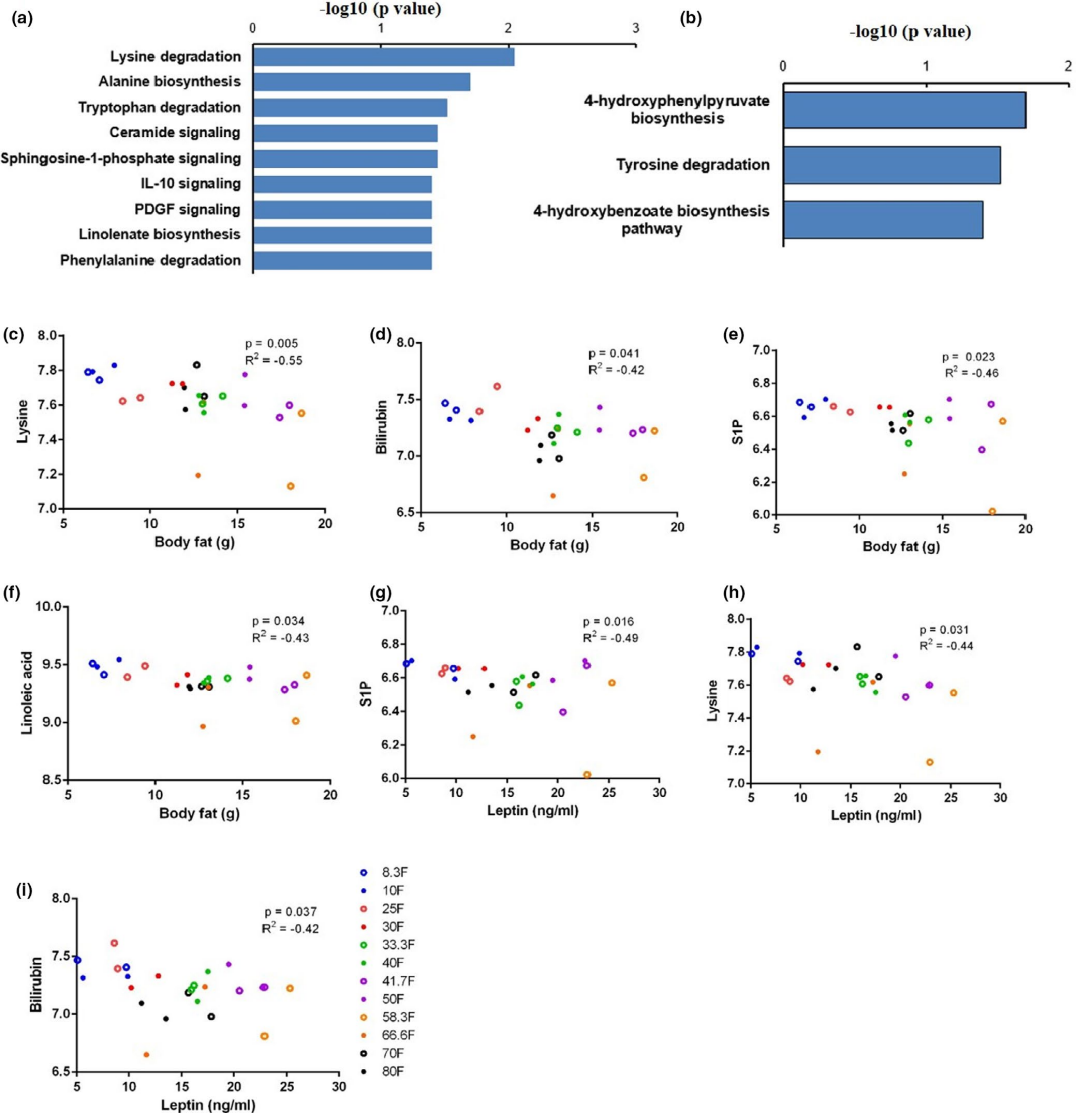
Significantly changed metabolic pathways and metabolites in the serum of mice fed different fat content diets. (a) Significantly changed metabolic pathways related to the increasing dietary fat content. (b) Significantly changed metabolic pathways correlated independently with the increasing fat level. (c‐i) The relationship between body fat, serum leptin concentration and S1P, linoleic acid, lysine, bilirubin, concentrations, respectively. Pearson correlation analysis was performed to analyze the correlation between serum metabolite expression levels and body fat, serum hormone concentrations

To further explore the significantly affected metabolite pathways under different fat content diets, we also performed the GLM analysis for all metabolites and then selected metabolites significantly correlated with dietary fat levels (*p* < 0.05) for analysis using the IPA software. Lysine degradation (*p* = 0.009), alanine biosynthesis (*p* = 0.02), tryptophan degradation (*p* = 0.03), and the IL‐10 signaling pathway (*p* = 0.04) were the pathways most affected by the dietary fat (Figure 6a) (Table S8). There were 2/14 and 2/25 metabolites significantly changed in lysine degradation pathway and tryptophan degradation pathway, respectively. Whereas only 1 metabolite showed significant differential expression in alanine biosynthesis (1/2) and IL‐10 signaling (1/4) pathway, respectively. Apart from these significantly changed metabolic pathways, Pearson correlation with dietary fat content respectively under 10% protein and 25% protein conditions (808 metabolites) also showed ceramide signaling (*p* = 0.036), sphingosine‐1‐phosphate signaling (S1P) (*p* = 0.036), PDGF signaling (*p* = 0.042) in 25% protein group and linolenate biosynthesis (*p* = 0.04), phenylalanine degradation (*p* = 0.043) pathway under 10% protein condition were all significantly affected pathways with the elevation of dietary fat level (Figure 6a) (Table S8). Of 808 metabolites, 613 metabolites were correlated both with dietary fat and carbohydrate content and 226 metabolites were associated both with protein and fat content in the diet (Figure 5a, b). There were 130 metabolites had independent significant correlations with dietary fat content, and the following pathway analysis of these 130 metabolites indicated that 4‐hydroxyphenylpyruvate biosynthesis (*p* = 0.02), tyrosine degradation (*p* = 0.03), and 4‐hydroxybenzoate biosynthesis pathway (*p* = 0.04) were significantly changed pathways with increasing dietary fat content (Figure 6b). We also performed Pearson correlation between metabolites in significantly affected pathways by dietary fat and physiological traits. We found lysine, bilirubin, sphingosine‐1‐phosphate, linoleic acid all had significant negative correlations with body fat mass (Figure 6c‐6f), whereas only lysine, sphingosine‐1‐phosphate, and bilirubin were significantly related to the serum leptin concentration (Figure 6g‐6i) (p and r values in Table S10). There were no metabolites in significantly altered metabolic pathways that had significant correlation with serum insulin level.

There were 995 metabolites significantly correlated with dietary carbohydrate content under Pearson correlation (Figure 5a). The most affected pathways by carbohydrate level included lysine degradation (*p* = 0.02), tRNA charging (*p* = 0.03), alanine biosynthesis (*p* = 0.03), and iNOS signaling pathway (*p* = 0.04) (Table S9).

### 2.4 Relationships between circulating metabolites and hepatic gene expression

We performed correlations between metabolites in significantly changed metabolic pathways with the increase of dietary protein content and genes involved in amino acid, fatty acid metabolism, and gluconeogenesis. The amino acid metabolism gene *Got1* and amino acid transport gene *Slc43a1* were significantly negatively related to circulating levels of several amino acids (Figure S7d, S7e) (p and r values in Table S11). Genes involved in gluconeogenesis *G6pc* and *Pck1* were negatively associated with alanine, arginine, histidine, leucine, lysine, and serine (Figure S7f) (p and r values in Table S11). Ornithine, involved in the citrulline biosynthesis pathway, had significant positive associations with amino acid metabolism and transport genes *Gpt* (*p* = 0.024), *Prodh* (*p* = 0.006), *Slc43a1* (*p* = 0.02), *Slc7a2* (*p* = 0.036) and gluconeogenesis gene *Pck1* (*p* = 0.02) (Figure S7d‐f).

### 2.5 Discussion

The current data on changes in hepatic gene expression in response to changes in dietary composition, where we detected changes in more than 4000 genes in response to each dietary component, contrasts enormously with a previous study which involved C57BL/6 mice exposed to 25 different diets and which indicated dietary protein intake was most powerful driver of hepatic gene expression (leading to correlated changes in 1279 genes), and that there were only 8 and 3 genes significantly correlated with changes in dietary carbohydrate and fat intake, respectively (Gokarn et al., 2018). There are several potential reasons for the differences between our work and this previous study. We used the Illumina NextSeq 500 sequencer for RNA‐sequencing whereas the previous study measured the gene expression by using less sensitive Affymetrix arrays. Further, the previous study analyzed livers of 46 mice fed one of 25 diets which means some diets were represented by a sample of just one mouse. In contrast with 68 samples across 12 diets, most of the diets in our study were represented by 4 independent replicates. This difference leads us to have greater power to detect differences in gene expression. That power could translate to an increase in the number of genes detected as significant for fat and carbohydrate, but not protein, if the effect sizes of such genes were greater, or if there was greater individual variability in response to fat and carbohydrates in the diet than there is to dietary protein. In addition, the age of onset and makeup of the other dietary components were also different. Perhaps most importantly the mice in the previous study were still growing which may have placed a premium on changes related to protein intake, while in the present study the mice were already mature at the onset of dietary manipulation and 8 months old when measured (Gokarn et al., 2018).

We detected specific gene expression patterns in key metabolic pathways. Several fatty acid synthesis genes were upregulated in the liver with the increase of dietary protein content both in high‐fat and low‐fat groups. In addition, most of the amino acid metabolism and transport genes also had higher expressions in the higher dietary protein intake group, but very few of these genes were correlated with serum insulin, leptin concentration, and dietary fat intake. A previous study also indicated that a high protein diet (45% of energy) induced higher expression of several amino acid metabolism and uptake genes, of which *Got1*, *Gpt*, and *Slc43a1* were also indicated in our study. They also found fatty acid synthesis gene *Gnpat* was upregulated in the high protein group although there was no body fat gain (Díaz‐Rúa et al., 2017). In another study, the liver glutaminase gene *Gls2* was one of the most affected gene by the dietary protein intake, but was unaffected by dietary carbohydrate and fat intake (Gokarn et al., 2018; Miller et al., 2018; Okun et al., 2021). In contrast in our study, *Gls2* expression was significantly changed both by dietary protein and carbohydrate intakes.

Several recent studies showed *Fgf21* was upregulated by a low protein diet (Laeger et al., 2014, 2016). It has been indicated that *Fgf21* had highest expression under combination of low protein and high carbohydrate intakes (Solon‐Biet et al., 2016). However, in our study, there was no significant correlation between *Fgf21* expression and dietary carbohydrate intake. Though the *Fgf21* expression was differentially expressed between different protein content groups (5% protein had significant higher expression compared to the 25% protein group both 60% fat and 20% fat conditions), there was no significant differential expression between 35% carbohydrate (60% fat) and 75% carbohydrate (20% fat) group under 5% protein. That is, *Fgf21* expression was not dependent on the dietary carbohydrate level or intake. The difference between these two studies may related to the dietary component, we fixed fat levels when we investigated the carbohydrate impact on gene expression, whereas in the previous study they did not fix any of the macronutrients.

Circulating levels of several amino acids were decreased as the dietary protein content increased. We also found that several amino acid‐tRNA ligase gene expressions were also decreased in the higher dietary protein groups. This suggests amino acids were potentially being utilized in lipid synthesis and glucose synthesis, and as expected from this hypothesis, genes for the gluconeogenesis enzymes *G6pc* and *Pck1* were both upregulated with the increase of dietary protein levels. This is consistent with a previous study which indicated that during short‐term fasting female mice used amino acids to synthesize glucose and lipids (Della Torre et al., 2018).

Apart from the tRNA charging pathway, the EIF2 signaling pathway, p70S6K signaling pathway, and mTOR signaling pathways were significantly altered with the increase of dietary protein level. It has been previously suggested that the expression level of Mtor was increased in lower protein intake, whereas in our study Mtor itself (as opposed to the whole pathway) had no significant correlation with both protein and carbohydrate intake, this may linked to the differences between two studies mentioned in the first paragraph. EIF2 is a highly conserved signal regulating cell responses to a variety of stresses, so the dysregulation of this pathway has been linked to many human diseases. EIF2 signaling has also been identified as a low protein or low amino acid sensing pathway, and previous studies have shown it was activated by low dietary protein (Guo & Cavener, 2007; Laeger et al., 2016; Maida et al., 2016; Wu et al., 2021). This effect was consistent with our data. The important downstream molecule *Atf4* had higher expression in 5% low protein group through *Atf4* was not significantly correlated with the dietary protein level and protein intake if plotted with the 6 different levels of protein. Nevertheless, most of the genes (84/95) in EIF2a pathway were negatively correlated with the dietary protein intake. The unfolded protein response (UPR) is a cellular stress response related to the endoplasmic reticulum, dysregulation of this process has been implicated in many diseases such as type II diabetes and cancer (Jovaisaite et al., 2014; Walter & Ron, 2011), and this pathway was also one of the most significantly affected pathways with the increasing of dietary protein contents. The transcription factors NFIX and Sox13 that were mostly enriched in the promoters of genes significantly correlated with dietary macronutrient have been indicated to be involved in the regulation of oxidative stress and glycolysis, respectively (Cui et al., 2020; Liu et al., 2020; Saleem et al., 2020).

In most affected serum metabolic pathways, apart from tRNA charging and amino acid degradation pathways, citrulline biosynthesis, methylglyoxal degradation, and acetone degradation pathway were also significantly changed. Arginase metabolizes the hydrolysis of arginine into ornithine and urea, whereas NOS can degrade arginine into citrulline (Husson et al., 2003; Jobgen et al., 2006; Luiking et al., 2010; Sailer et al., 2013). This process might lead to vascular endothelial dysfunction in the early stage of obesity (Ito et al., 2018), so arginine degradation with the increase of dietary protein content in our study may be one of the mechanisms of slight increase in body fat, but this mechanism needs further investigation. Methylglyoxal degradation and acetone degradation pathways were likely the result of methylglyoxal degraded into acetone. Early studies showed that methylglyoxal caused type II diabetes and oxidative stress, and so methylglyoxal was identified as a major therapeutic target for type II diabetes (Dornadula et al., 2015; Hanssen et al., 2019; Yılmaz et al., 2017), whereas in our results methylglyoxal was negatively related to the body fat gain.

Several metabolic pathways were significantly changed in response to varying contents of fat. A previous study indicated that oral alanine administration improved glucose tolerance in both chow diet and high‐fat diet‐treated mice (Adachi et al., 2018). In our study, alanine was also significantly decreased as dietary fat content increased. It will be interesting to investigate alanine effects on other metabolic parameters under different nutritional environments. IL‐10 signaling and linoleic acid signaling were also identified as the significantly changed pathways in response to dietary fat. In a recent study, it has been showed that ablation of IL‐10 improved insulin sensitivity and inhibited diet‐induced obesity (Rajbhandari et al., 2018), also in another study, IL‐10 was indicated to be decreased in childhood obesity (Liu et al., 2018). Consistent with many previous studies, activity of the linoleic acid pathway was decreased as dietary fat content increased (Caligiuri et al., 2013; Cedernaes et al., 2013). It has been indicated in another study that linoleic acid regulation of glucose homeostasis in obesity was dependent on the sex difference (Kowalski et al., 2013; Zhuang et al., 2018).

As for the S1P metabolic pathway, there has been controversy about how S1P signaling was changed under different nutritional states. It was indicated in one study that plasma S1P is elevated in obesity (Kowalski et al., 2013), and sphingosine kinase 2 knockout mice were protected from obesity and insulin resistance (Ravichandran et al., 2019). However, there were several studies indicating that S1P was associated with beneficial effects of caloric restriction in male Wistar rats and C57BL/6 mice and lifespan regulation in mammals (Babenko & Shakhova, 2014; Collino et al., 2013; Lightle et al., 2000). In the present study, we found that S1P signaling was decreased with the elevation of fat content, which is opposite the effect of graded levels of caloric restriction which showed S1P was upregulated as the caloric restriction level increased (Green et al., 2017). As S1P was identified as significantly changed metabolite in both graded levels of caloric restriction and different fat content diet treatment studies, it seems to be a strong potential target linking nutrition and aging. The significantly changed gene pathway in response to varying contents of fat, Nrf2 signaling pathway was indicated in several studies that related to the oxidative stress and aging (Tyshkovskiy et al., 2019; Zhang et al., 2015).

Dietary restriction extends lifespan across multiple species (Ingram & de Cabo, 2017; Mercken et al., 2012). Previous work showed that hepatic gene expression levels in major aging‐related pathways (IGF‐1, NF‐kB, and mTOR) were altered under restriction in a manner consistent with increased lifespan (Derous et al., 2017). In the insulin/IGF‐1 signaling pathway, *Igf1* and *Insr* were negatively correlated with the caloric restriction level, and also increased with the increase of protein intake in the present study, but *acly* that had strong negative correlation with the level of caloric restriction had almost no correlation with protein intake. In the NF‐KB signaling pathway, NF‐kB/ReIB complex also had almost no correlation with protein intake but its expression was increased with the increase of caloric restriction level. In the mTOR signaling pathway, the number of genes that significantly correlated with dietary protein, fat, and carbohydrate intake were 5, 10, and 4, respectively. Overall, many more aging‐related genes had significant correlations with dietary fat intake than protein and carbohydrate intake. Moreover, the pattern of gene expression changes in relation to dietary fat intake was in the opposite direction to the effect of graded levels of caloric restriction. Compared to a recent study that indicated five key nutrient sensing genes linked nutrient with aging (*Mtor*, *Fgf21*, *Igf1*, *Sitr1*, *Prkab2*), only one of these genes (*Prkab2*) was significantly correlated both with changing levels of dietary protein and fat content. In conclusion, intake of fat appeared to have more significant effect on aging‐related gene expression as more genes in aging‐related pathways (notably mTOR, IGF‐1, and NF‐KB) had significant correlations with dietary fat intake than protein and carbohydrate intake.

### 2.6 Limitations of the study

One strength of this work is that the mice were exposed to the different diets for a protracted period, meaning they had a long time to respond to the intervention. However, in a sense that strength can also be a weakness because the impacts may not only be direct effects of the diets but downstream impacts of the diets on other features such as adiposity. At the moment, we cannot separate these possibilities.

## 3 EXPERIMENTAL PROCEDURES

### 3.1 Ethical statement

All animal procedures were reviewed and approved by the Institute of Genetics and Developmental Biology Chinese Academy of Sciences.

### 3.2 Mice and experimental diet

Data in the current paper pertain to mice involved in a large dietary manipulation experiment, some aspects of which have already been published. These previous publications have included patterns of body weight, adiposity, and hypothalamic gene expression (Hu et al., 2018, 2020b) and glucose homeostasis (Hu et al., 2020a). All procedures in this study were reviewed and approved by the Institutional Review Board, Institute of Genetics and Developmental Biology, Chinese Academy of Sciences. We previously exposed C57BL/6 mice to a total of 29 different diets varying from 8.3 to 80% fat, 10 to 80% carbohydrate, 5 to 30% protein, 5 to 30% sucrose content and found only increased dietary fat content was associated with elevated energy intake and adiposity but not the protein or carbohydrate content. Whereas in the current study, we investigated the effects on mouse hepatic gene expression (by RNA‐seq) of *ad libitum* intake of six different levels of dietary protein (5 to 30%) combined with both high‐fat (60%) and low‐fat (20%) conditions, leading to 12 different levels of dietary carbohydrate (10% to75%). In addition, we explored the impacts of 24 different diets (varying from 5% to 30% protein, 8.3% to 80% fat, and 10% to 80% carbohydrate) on serum metabolite levels by untargeted metabolomics. Full details of diets are in supplementary Table S12–15). During the whole experimental period, mice were singly housed under controlled 22–24 ℃ temperature and 12:12 light dark cycle conditions. Mice were killed by rising concentrations of CO_2_ for the collection of tissues and serum, which were quickly snap frozen in liquid nitrogen and then stored in a −80℃ freezer until analysis. More information about procedures and experimental designs can be found in our previous papers (Hu et al., 2018, 2020b).

### 3.3 Liver RNA extraction and transcriptome analysis

The RNA of 68 individual mice (n = 5–6 mice per group) was extracted from liver tissues and sent to Beijing Genomic Institute (BGI) for RNA‐sequencing. Liver tissues were put in Trizol (Invitrogen) reagent and homogenized by Bead Ruptor (OMNI), then total RNA were extracted by chloroform/isoamyl alcohol/RNA precipitation solution (1.2 M NaCl & 0.8 M disodium hydrogen citrate sesquihydrate) step by step and purified by 75% ethanol. Each sample was sequenced by 75 bp long reads from paired ends. The raw data of RNA‐seq were analyzed using the method described in the previous study (Wu et al., 2021). Normalized counts were used to express the specific gene expression level, to explore significantly correlated pathways, respectively, under 60% fat and 20% fat conditions, Pearson correlation analysis was used for normalized counts of all genes by using correlation method in R‐3.5.3, and then significantly correlated genes (*p* < 0.05), respectively, under 60% fat and 20% fat condition were analyzed by IPA (www.ingenuity.com) software to obtain significantly affected pathways (*p* < 0.05), p‐values for each correlation were adjusted using the Benjamini–Hochberg procedure using a false discovery rate of 5%.

### 3.4 Serum metabolite measurement and analysis

From each diet group, 12 serum samples from 12 individual mice were collected. Six samples were pooled together as one sample, resulting in 2 pooled samples in each diet group (n = 48 samples across 24 diets). Serum metabolites were extracted by mixing Chloroform: Methanol: Serum in 1:3:1 ratio and following centrifuged at 1,3000 rpm for 3 minutes, supernatant was collected and did LC‐MS using an OrbitrapTM ExactiveTM mass spectrometer at the Glasgow Polyomics facility. Each metabolite was expressed by raw peak intensities at last, and then, these peaks were analyzed step by step using R packages according to the method described in the previous study (Wu et al., 2021). Generalized linear modeling and Pearson correlation were performed for normalized intensities of each metabolite across the different protein level and selected the significantly correlated metabolites (*p* < 0.05) into the IPA software to get the most affected pathways.

### 3.5 Hormone measurements

Liver triglyceride and glycogen concentrations were measured by using glycogen assay kit (#KA0861, Abnova) and general triglyceride ELISA Kit (EK3875, SAB), respectively. All procedures were performed according to the manufacturers’ instruction.

### 3.6 CiiiDER analysis

CiiiDER software was downloaded from CiiiDEr.org with the M. musculus GRCm38.94 genome files. Transcription factor analysis was performed on promoter regions spanning +1500 to −500 bp from the predicted transcription start site (Gearing et al., 2019). The background gene lists were the genes had no significant correlations with dietary macronutrient composition.

### 3.7 Correlation analysis with physiological traits

Mean body fat, macronutrient intakes, and serum hormones measured in the last week of dietary manipulation were correlated (Pearson's correlation) with gene normalized counts and normalized metabolite intensities for each sample. All Pearson correlations (*P*‐*values*) in this study were adjusted using the Benjamini–Hochberg procedure using a false discovery rate of 5%.

### 3.8 Generalized linear model

We analyzed data on specific hepatic gene expression and serum metabolite using generalized linear modeling (GLM) with gene expression as the dependent variables and dietary levels of fat and protein, and the interactions of the two macronutrients as the independent predictors. To explore the significantly affected gene and metabolite pathways, we also performed the GLM analysis for all genes and metabolites then selected genes and metabolites significantly correlated with dietary protein or fat content (*p* < 0.05) into the Ingenuity pathway analysis (IPA) software.

## CONFLICT OF INTEREST

The authors declare no competing interests.

## AUTHOR CONTRIBUTIONS

Y.G.W conducted the experiments, analyzed the data, and co‐wrote the manuscript. J.R.S. directed the project, conceived and designed the experiments, contributed to the analysis, and co‐wrote the paper. D.B.Y., B.G.L., L.L., G.L.W., L.W., M.L., J.B.L., S.M.H., C.Q.N., X.Y.X., and Y.C.X. contributed to the data collection. G.L.W., A.D., G.C., and D.D. contributed to RNA‐seq, metabolomics raw data, and IPA‐related analysis. All authors approved the final version prior to submission for publication.

## Supporting information

Supplementary Material

## Data Availability

The data that support the findings of this study are available from the authors upon request. The metabolomics and transcriptomics data have been uploaded on Mendeley and relevant DOI is as follows: 10.17632/2s5ccsspfk.2. I confirm that I have included a citation for available data in my references section.
