# Supplementary material for: Effects of dietary macronutrients on the hepatic transcriptome and serum metabolome in mice
Source: Aging Cell. 2022 Mar 10;21(4):e13585. doi: 10.1111/acel.13585 (PMC9009132; doi:10.1111/acel.13585)
Supplement: Supplementary file 1 — Supplementary Material [file ACEL-21-e13585-s001.pdf]

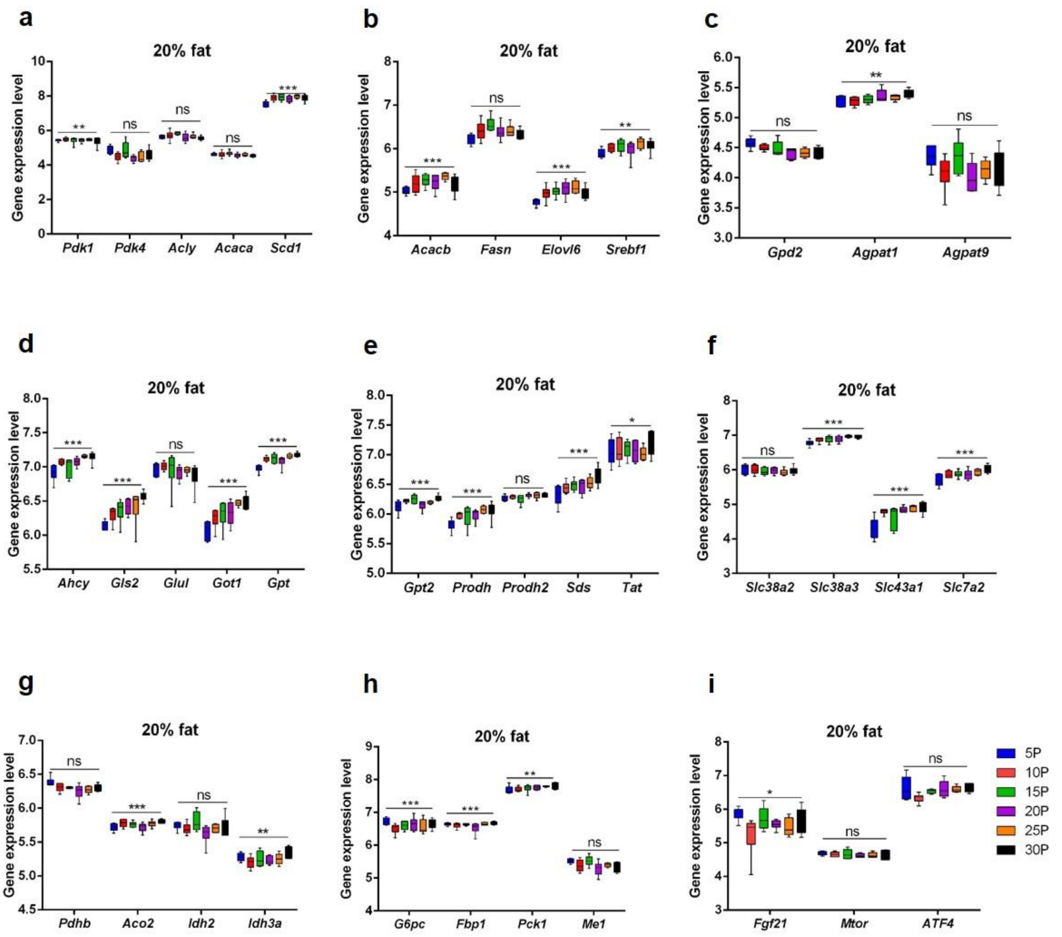

Figure S1. The gene expression levels in fatty acid and amino acid metabolism pathway in the livers of mice treated with variable protein content and fixed 20% fat diets. (a and b) Fatty acid synthesis metabolism, (c) triglyceride synthesis metabolism, (d-e) amino acid metabolism, (f) amino acid transport metabolism, (g) TCA cycle, (h) gluconeogenesis metabolism and (i) regulation of protein intake related genes (n = 5-6). Generalized linear modelling was performed to analyze the dietary protein effect on specific gene expression. * $p < 0.05$, ** $p < 0.01$, *** $p < 0.001$, ns $p > 0.05$. Values are represented as mean $\pm$ SD.

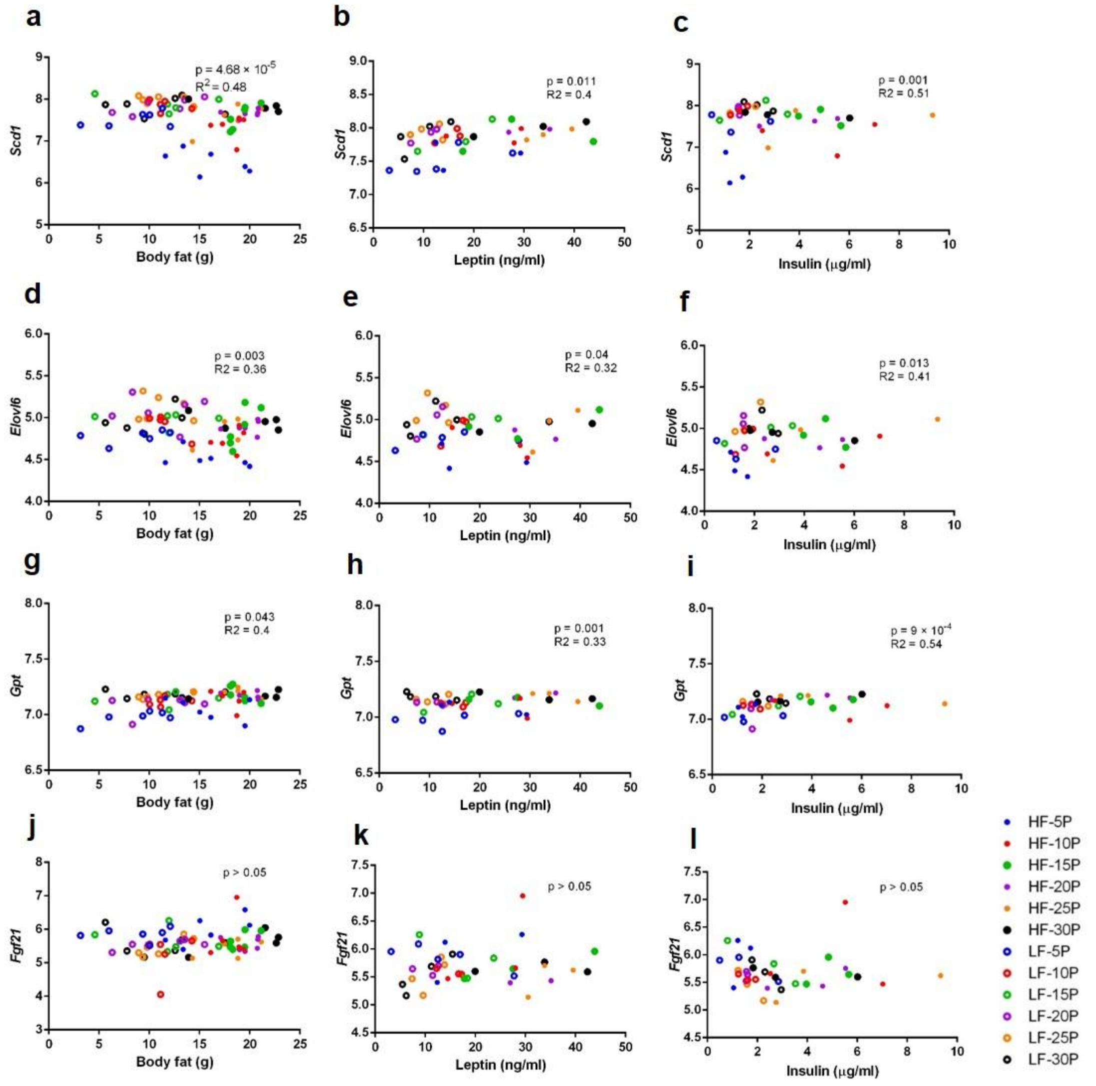

Figure S2. The correlation between body fat, serum hormone concentrations and gene expression levels. The relationship between body fat, leptin, insulin concentration and (a-c) *Scd1*, (d-f) *Elovl6*, (g-i) *Gpt*, (j-l) *Fgf21*. Pearson correlation analysis was performed to analyze the correlation between gene expression levels and body fat, serum hormone concentration.

a

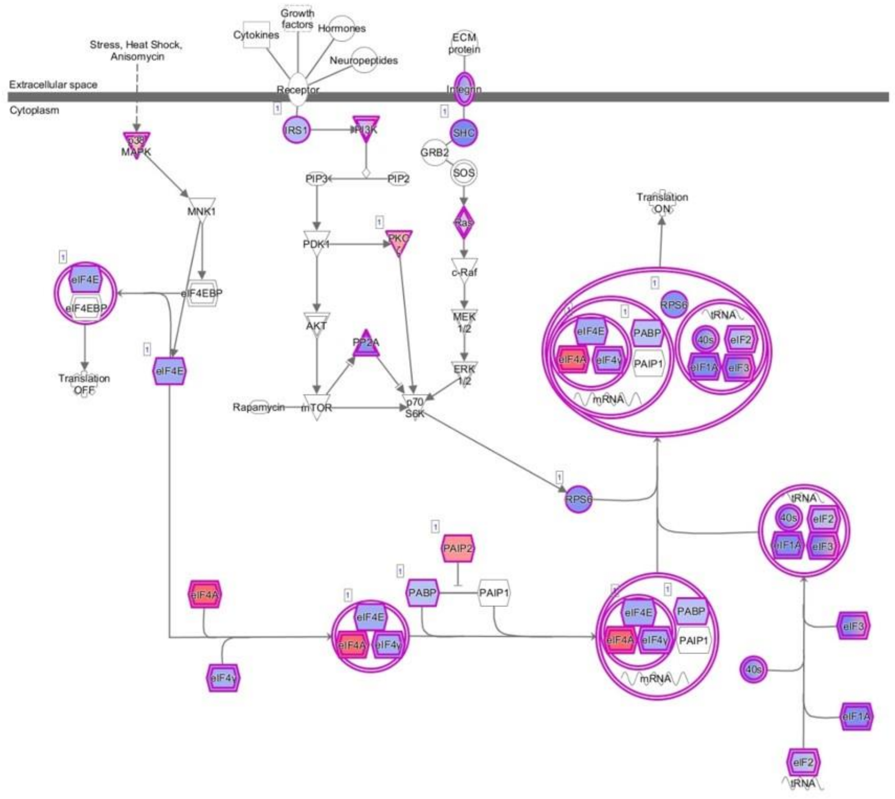

© 2000-2020 QIAGEN. All rights reserved.

b

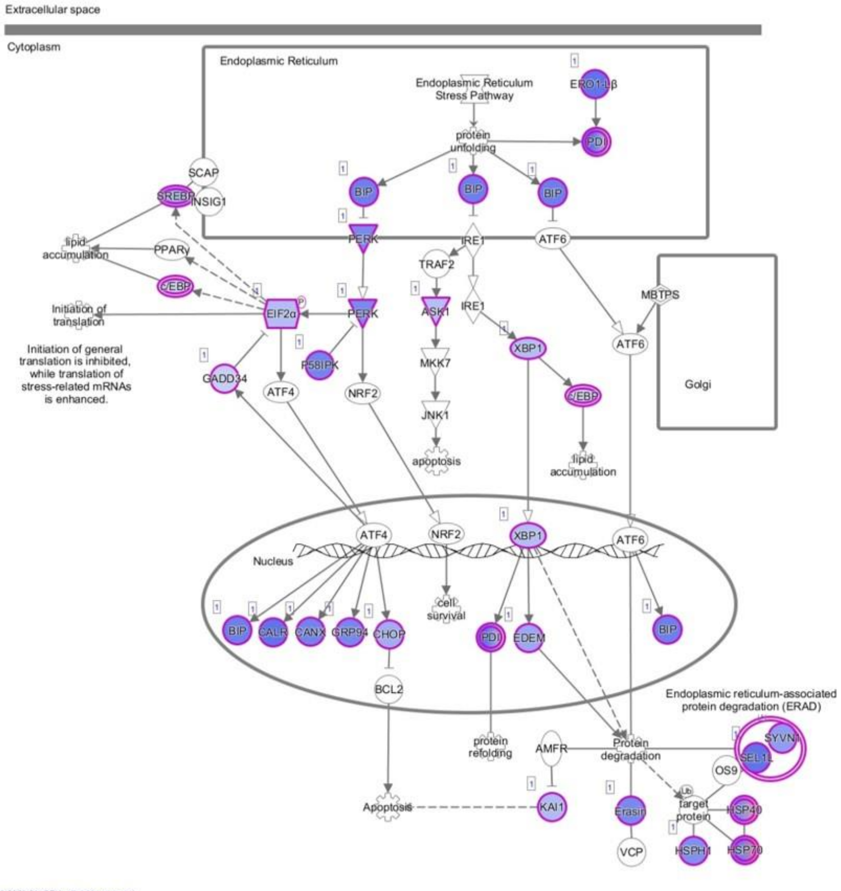

© 2000-2020 QIAGEN. All rights reserved.

Figure S3. Pathway diagram showing GLM regression of gene expression against dietary protein contents. (a) Regulation of eIF4 and p70S6K signaling, (b) unfolded protein response pathway diagram, red indicates positive and blue indicates the negative regression with the protein content in the diet, gray indicates no significance. Generalized linear modeling was performed to analyze the dietary fat effect on gene expression.

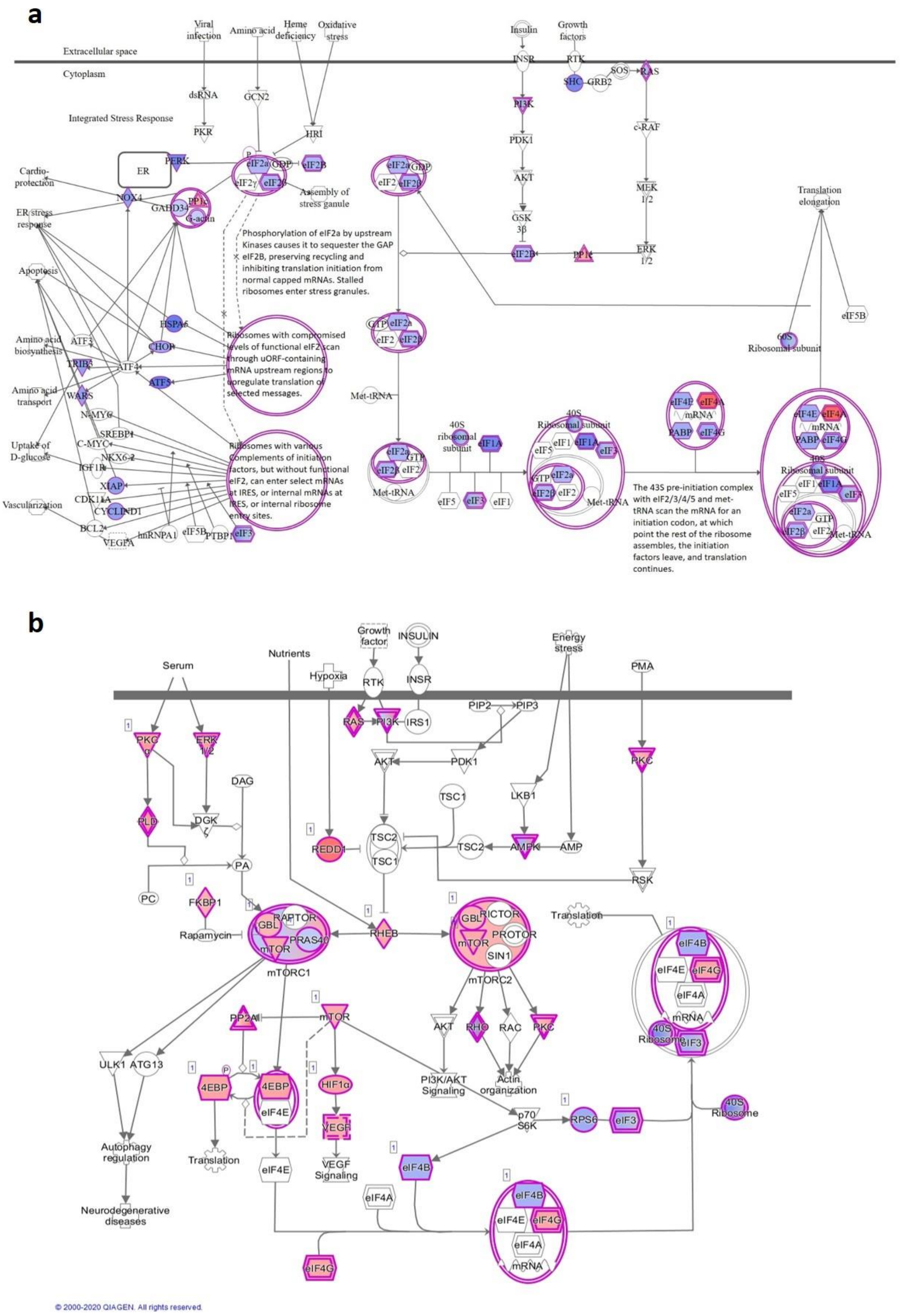

Figure S4. Pathway diagram showing GLM regression of gene expression against dietary protein and fat contents. (a) eIF2 signaling pathway, (b) mTOR signaling

pathway diagram, red indicates positive and blue indicates the negative regression with the protein content in the diet, gray indicates no significance. Generalized linear modeling and Pearson correlation were performed to analyze the dietary fat effect on gene expression.

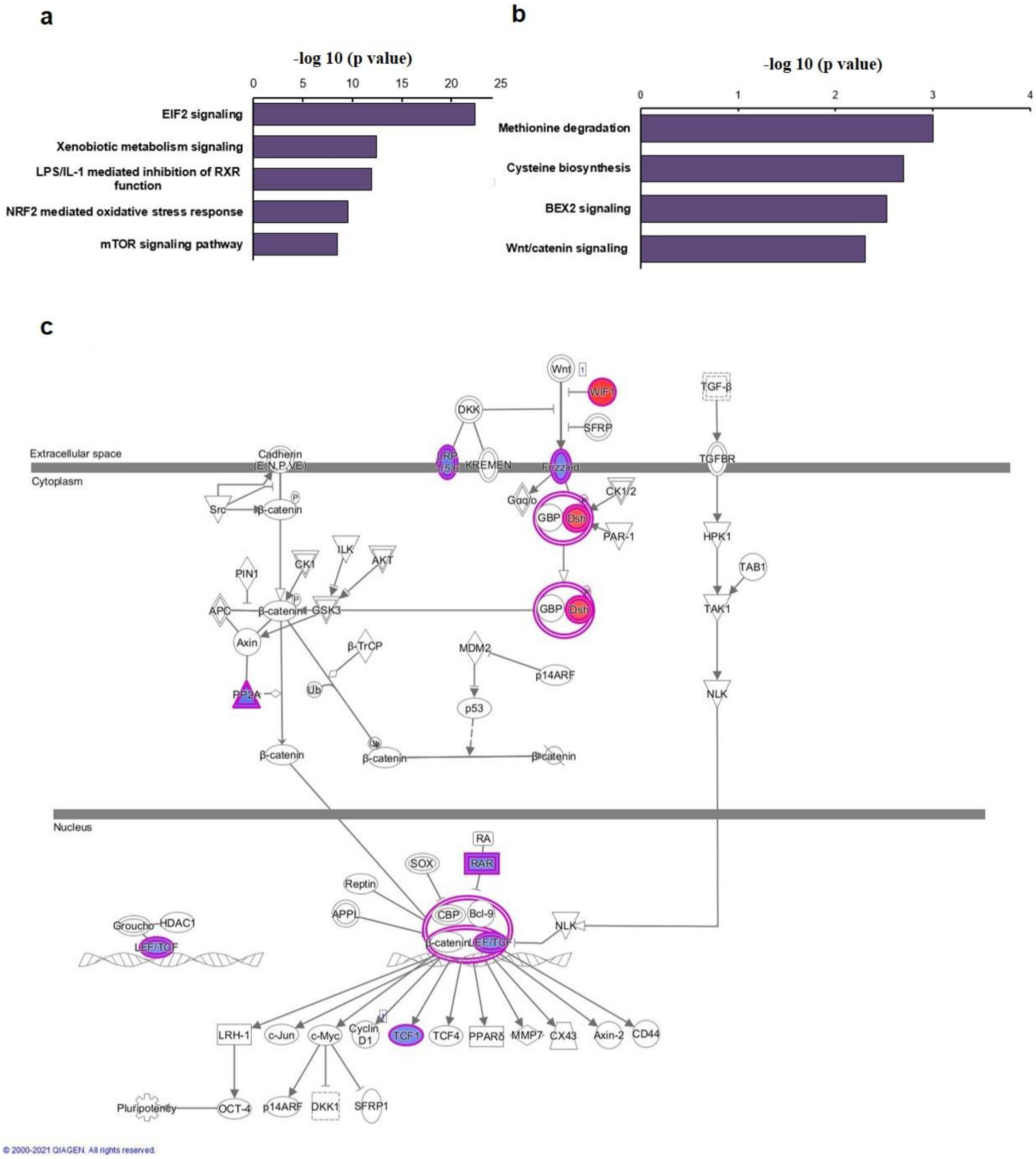

Figure S5. Significantly changed gene pathways with increasing carbohydrate content. (a) Significantly changed gene pathways related to the increasing dietary carbohydrate content. (b) Significantly changed gene pathways correlated independently with the increasing carbohydrate level. (c) Wnt/catenin signaling pathway, red indicates positive and blue indicates the negative regression with the protein content in the diet, gray indicates no significance. Generalized linear modeling and Pearson correlation were performed to analyze the dietary carbohydrate effect on gene expression.

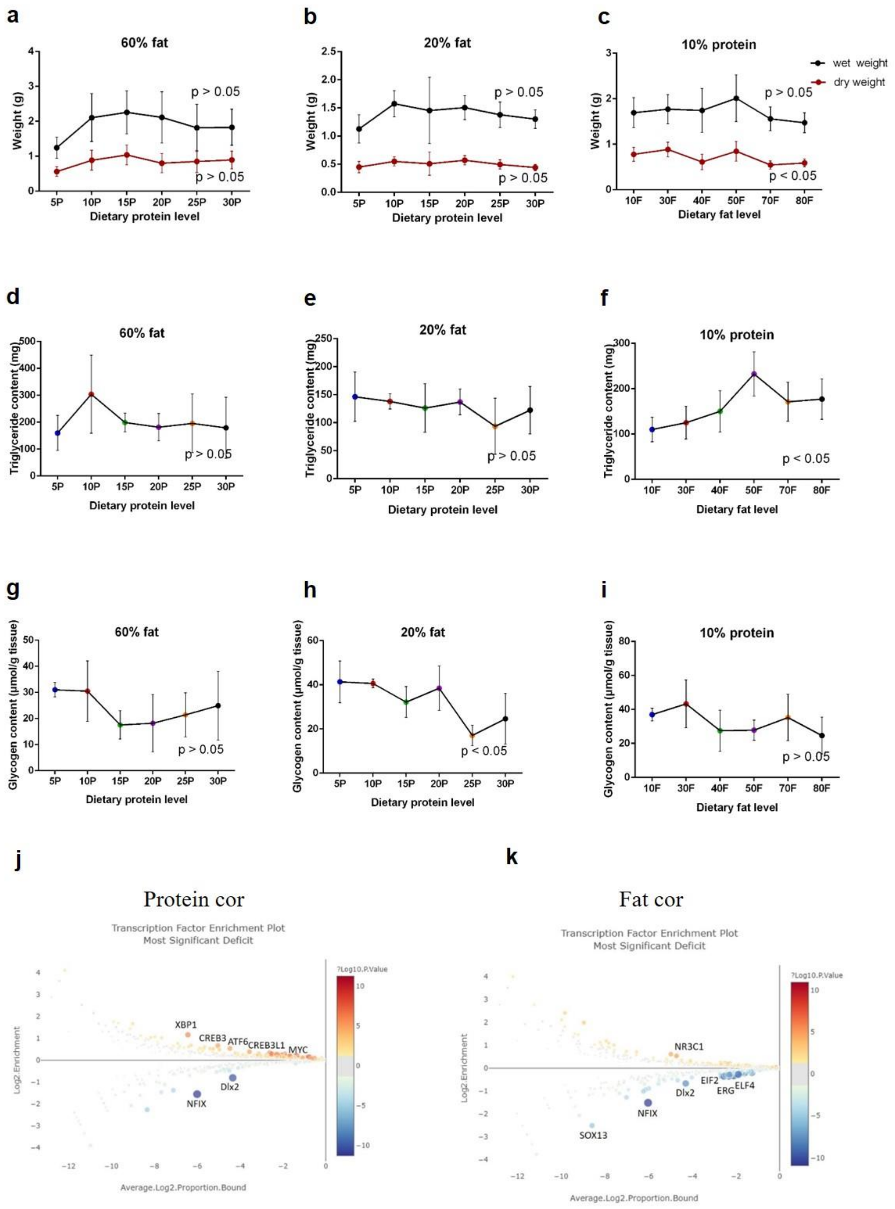

Figure S6. The liver physiological parameters of mice treated with variable macronutrient content diets and significantly enriched transcription factors. The wet and dry liver weights of mice fed with different protein content diets under (a) 60% fat, (b) 20% fat and (c) different fat content diets under 10% protein condition. The liver triglyceride and glycogen concentrations of mice fed with different protein content diets under (d, g) 60% fat, (e, h) 20% fat and (f, i) different fat content diets under 10% protein condition. (a and b) Significantly enriched transcription factors in the promoters of genes significantly correlated with dietary protein content (j) and fat (k) content. Pearson correlation analysis was performed to analyze the dietary macronutrient effect on liver physiological data. Values are represented as mean ± SD.

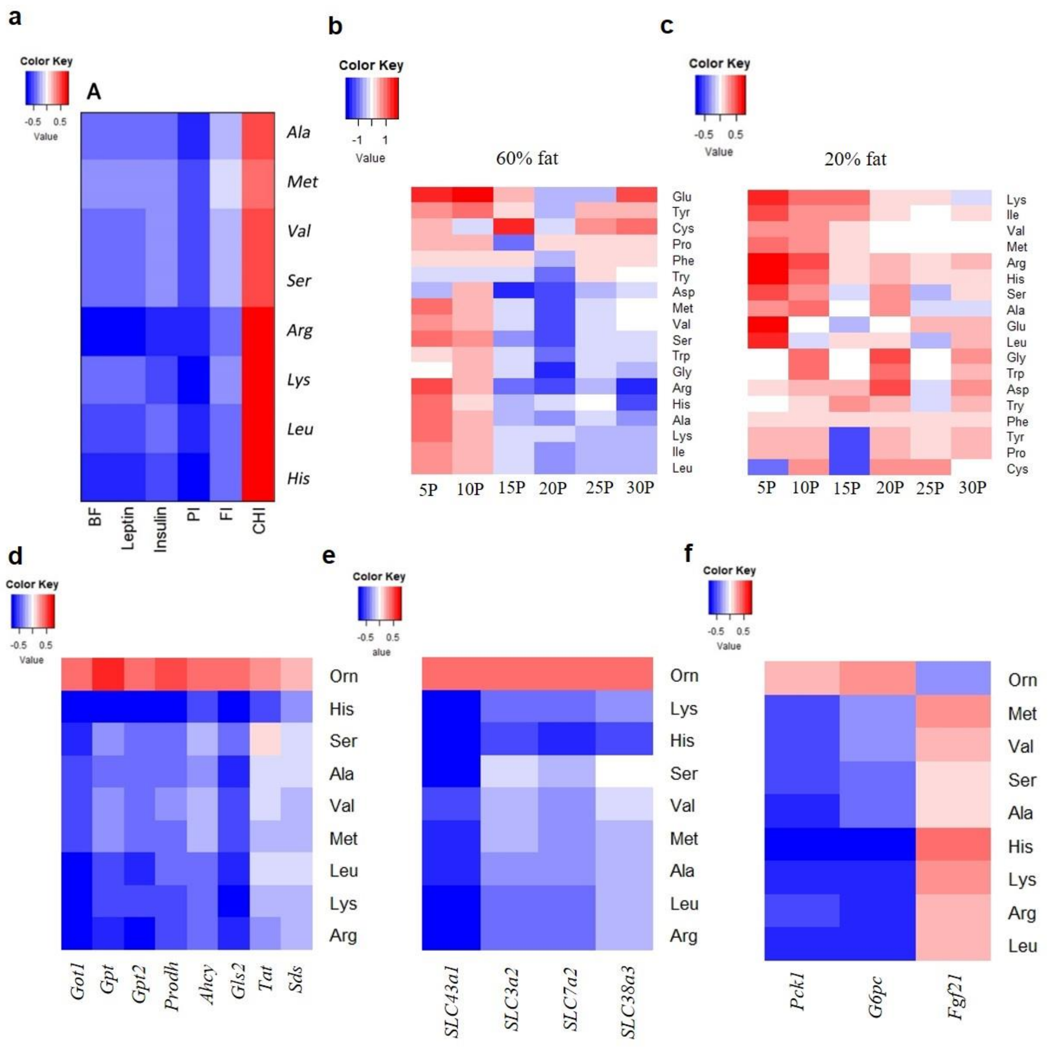

Figure S7. The relationship between physiological data, gene expression levels and significantly changed metabolites. (a) The correlations between body fat, serum leptin, insulin concentration, protein intake (PI), fat intake (FI), carbohydrate intake (CHI) and several amino acids concentration. (b-c) Log-transformed concentrations of several amino acids in different protein content diet treatment groups respectively. (d-f) The relationship between significantly changed metabolites with increasing protein content and genes involved in (d) amino acid metabolism, (e) amino acid transport pathway and (f) gluconeogenesis process respectively. Pearson correlation analysis was used for analysis.

Table S1. Significantly correlated gene pathways with dietary protein content.

Pathway	Protein (4005 genes)			Protein (1886 genes)		
	Up	Down	*P*-value	Up	Down	*P*-value
EIF2 signaling	2.43%	45.80%	<0.001			
Protein ubiquitination pathway	8.12%	16.24%	<0.001			
tRNA charging	0%	60.52%	<0.001	0%	31%	<0.01
Unfolded protein response	3.63%	47.27%	<0.001			
TCA cycle	71.43%	0%	<0.001			
Colanic acid building blocks biosynthesis	7.14%	64.3%	<0.001			
Regulation of eIF4 and p70S6K signaling	4.45%	31.84%	<0.001			
mTOR signaling pathway	8.38%	23.56%	<0.001			
Amino acid metabolism	51.16%	48.84%	<0.001			
Protein synthesis	21.95%	54.27%	<0.001			
Nucleic acid metabolism	39.4%	60.6%	<0.001			
Sirtuin signaling pathway				8.06%	8.46%	<0.01
DNA methylation and transcriptional repression signaling				4.17%	29.17%	<0.01
Super-pathway of serine and glycine biosynthesis				14.29%	42.86%	<0.01

Significantly affected pathways from IPA software after performed generalized linear modelling analysis and Pearson correlation of all genes with dietary protein levels (from low protein to high protein). The percentage of genes up- and down-regulated for each pathway calculated using IPA. *P*-values are based on the number of genes found in liver samples relative to total number of molecules in known pathway.

Table S2. Significantly correlated gene pathways with dietary fat content.

	Fat (4232 genes)			Fat (546 genes)		
Pathway	Up	Down	*P*-value	Up	Down	*P*-value
Lipid metabolism	31.23%	0%	<0.001			
Nrf2 mediated oxidative stress response	41.34%	4.5%	<0.001			
Eif2 signaling	8.78%	34.15%	<0.001			
Xenobiotic metabolism	34.22%	3.8%	<0.001			
Cell morphology	40.52%	2.88%	<0.001			
LPS/IL-1 mediated inhibition of RXR function	33.17%	7.32%	<0.001			
mTOR signaling pathway	20.7%	20.2%	<0.001			
IL-15 signaling pathway				8.3%	0%	<0.05
Communication between innate and adaptive immune cells				5.06%	2.53%	<0.05
ERK/MAPK signaling pathway				4.52%	1%	<0.05

Significantly affected pathways from IPA software after performed Pearson correlation of all genes with dietary fat levels (from low fat to high fat). The percentage of genes up- and down-regulated for each pathway calculated using IPA. *P*-values are based on the number of genes found in liver samples relative to total number of molecules in known pathway.

Table S3. Significantly correlated gene pathways with dietary carbohydrate content.

Pathway	Carbohydrate (4292 genes)			Carbohydrate (272 genes)		
	Up	Down	*P*-value	Up	Down	*P*-value
EIf2 signaling	41.46%	7.32%	<0.001			
Xenobiotic metabolism	6.1%	31.93%	<0.001			
LPS/IL-1 mediated inhibition of RXR function	3.1%	33.66%	<0.001			
NRF2 mediated oxidative stress response	6.7%	33.52%	<0.001			
mTOR signaling pathway	23.23%	14.65%	<0.001			
Methionine degradation				7.7%	6.25%	<0.01
Cysteine biosynthesis				11.11%	5.56%	<0.01
BEX2 signaling				3.95%	2.63%	<0.01
Wnt/catenin signaling pathway				12.27%	3.07%	<0.01

Significantly affected pathways from IPA software after performed generalized linear modelling analysis and Pearson correlation of all genes with dietary carbohydrate levels (from low carbohydrate to high carbohydrate). The percentage of genes up- and down-regulated for each pathway calculated using IPA. *P*-values are based on the number of genes found in liver samples relative to total number of molecules in known pathway.

Table S4. The p-values and correlation coefficients of Pearson correlation results between genes in IGF-1 pathway and protein, fat, carbohydrate intakes.

	Protein intake		Fat intake		Carbohydrate intake	
Gene	**P value**	**R value**	**P value**	**R value**	**P value**	**R value**
Eif4e	0.004	-0.34	0.032	0.26		
Foxo3			0.016	0.29	0.0005	-0.41
Fyn	0.005	-0.34				
Gab1	0.003	0.36	0.015	0.3	0.0003	-0.42
Igf1	0.0003	0.43				
Irs1	0.045	-0.24				
Jun	0.013	0.3	4.6×10^{-7}	0.57	6.9×10^{-9}	-0.63
Shc1	5×10^{-6}	-0.52			0.007	0.32
Slc2a4	0.0004	0.42	4.3×10^{-5}	-0.47		
Acly			0.0001	-0.45	0.005	0.34
Eif4ebp1			0.0004	0.42		
Fos			4.7×10^{-9}	0.64	2.3×10^{-5}	-0.49
Mapk8			0.0003	0.42	0.01	-0.3
Mtor			0.003	0.36		
Pde3b			0.0004	0.42	0.0002	-0.43
Ptk2			0.001	0.38	0.012	-0.3
Ptpn1			0.02	0.28		
Sgk1			0.045	0.24		
Stat3			0.007	0.32	0.034	-0.25
Trip10					0.013	0.3

Table S5. The p-values and correlation coefficients of Pearson correlation results between genes in mTOR pathway and protein, fat, carbohydrate intakes.

	Protein intake		Fat intake		Carbohydrate intake	
**Gene**	**P value**	**R value**	**P value**	**R value**	**P value**	**R value**
Atg13	1.3×10^{-7}	-0.59			0.0001	0.45
Rps6	3.8×10^{-6}	-0.52	0.025	-0.27	4.4×10^{-7}	0.57
Eif4e	0.004	-0.34	0.032	0.26		
Ddit4	0.023	0.28	2.2×10^{-5}	0.49	1.6×10^{-7}	-0.58
Irs1	0.045	-0.24	0.0001	0.45		
Prkca						
Eif4ebp1			0.0004	0.42		
Mtor			0.003	0.36		
Fkbp1a			0.004	0.34		
Hif1a			0.011	0.31		
Rheb			0.011	0.3		
Eif4b			0.018	-0.28		
Akt1s1					0.034	0.26

Table S6. The p-values and correlation coefficients of Pearson correlation results between genes in NF-kB pathway and protein, fat, carbohydrate intakes.

	Protein intake		Fat intake		Carbohydrate intake	
Gene	**P value**	**R value**	**P value**	**R value**	**P value**	**R value**
Azi2	0.002	-0.37				
Bcl10	0.037	0.25	6×10^{-5}	0.47	0.0002	-0.43
Chuk	0.027	0.27	0.0009	0.4	0.0002	-0.43
Map3k14	0.017	-0.29				
Prkcz	0.02	0.28				
Tab1	0.009	0.31				
Tirap	0.008	-0.31			0.0002	0.43
Zap70	5.3×10^{-13}	0.74			4.4×10^{-6}	-0.52
Btrc			0.014	0.3		
Gsk3b			0.001	0.38	0.026	-0.27
Ikbkg			6.2×10^{-5}	0.47	0.008	-0.32
Mapk8			0.0003	0.42	0.01	-0.31
Peli1			0.001	0.38		
Relb			3.2×10^{-5}	0.48	0.005	-0.34
Tnfaip3			1.3×10^{-7}	0.59	0.0002	-0.43
Tnip1			0.001	0.38	0.048	-0.24
Ikbkg						

Table S7. Significantly correlated metabolic pathways with dietary protein content.

Pathway	Protein (732 metabolites)			Protein (362 metabolites)		
	Up	Down	*P*-value	Up	Down	*P*-value
tRNA charging	0%	18.6%	<0.001			
Arginine degradation	20%	20%	<0.001			
Alanine degradation	0%	100%	<0.001			
Citrulline biosynthesis	11.11%	11.11%	<0.01			
Lysine degradation	0%	15%	<0.01			
Methylglyoxal degradation	14.3%	14.3%	<0.01			
Acetone degradation	14.3%	14.3%	<0.01			
Histidine degradation	0%	20%	<0.05			
Glycine degradation				16.67%	16.67%	<0.01
Catecholamine biosynthesis				0%	25%	<0.01
Histamine degradation				0%	20%	<0.05
Dopachrome biosynthesis				0%	50%	<0.05

Significantly affected pathways from IPA software after performed generalized linear modelling analysis and Pearson correlation of all metabolites with dietary protein levels (from low protein to high protein). The percentage of metabolites up- and down-regulated for each pathway calculated using IPA. *P*-values are based on the number of metabolites found in serum samples relative to total number of molecules in known metabolic pathway.

Table S8. Significantly correlated metabolic pathways with dietary fat content.

	Fat (808 metabolites)			Fat (130 metabolites)		
Pathway	Up	Down	*P*-value	Up	Down	*P*-value
Lysine degradation	0%	14.3%	<0.01			
Alanine biosynthesis	0%	50%	<0.05			
Tryptophan degradation	0%	8%	<0.05			
Ceramide signaling	0%	25%	<0.05			
Sphingosine-1-phosphate signaling	0%	25%	<0.05			
IL-10 signaling	0%	25%	<0.05			
PDGF signaling	0%	16.7%	<0.05			
Linolenate biosynthesis	0%	14.3%	<0.05			
Phenylalanine degradation	14.3%	0%	<0.05			
4-hydroxyphenylpyruvate biosynthesis				0%	25%	<0.05
Tyrosine degradation				14.3%	0%	<0.05
4-hydroxybenzoate biosynthesis				9.1%	0%	<0.05

Significantly affected pathways from IPA software after performed generalized linear modelling analysis and Pearson correlation of all metabolites with dietary fat levels (from low fat to high fat). The percentage of metabolites up- and down-regulated for each pathway calculated using IPA. *P*-values are based on the number of metabolites found in serum samples relative to total number of molecules in known metabolic pathway.

Table S9. Significantly correlated metabolic pathways with dietary carbohydrate content.

Pathway	Carbohydrate (995 metabolites)			Carbohydrate (238 metabolites)		
	Up	Down	*P*-value	Up	Down	*P*-value
Lysine degradation	14.3%	0%	<0.05			
tRNA charging	7%	0%	<0.05			
Alanine biosynthesis	50%	0%	<0.05			
INOS signalling pathway	33.3%	0%	<0.05			

Significantly affected pathways from IPA software after performed generalized linear modelling analysis and Pearson correlation of all metabolites with dietary carbohydrate levels (from low carbohydrate to high carbohydrate). The percentage of metabolites up- and down-regulated for each pathway calculated using IPA. *P*-values are based on the number of metabolites found in serum samples relative to total number of molecules in known metabolic pathway.

Table S10. P and correlation coefficients of correlation analysis between expression levels of gene, metabolite and physiological data.

Gene	Protein	Body fat	Leptin	Insulin	Protein intake	Carbohydrate intake
Ahcy	1.87×10^{-16}					
Gls2	2.28×10^{-6}					
Got1	6.84×10^{-10}					
Gpt	2.98×10^{-10}	0.043，0.4	0.001，0.33	9×10^{-4}, 0.54		
Gpt2	1.01×10^{-8}					
Prodh	2.95×10^{-17}					
Sds	7.65×10^{-7}					
Tat	0.015					
Slc38a3	5.54×10^{-9}					
Slc3a2	1.37×10^{-7}					
Slc43a1	2.84×10^{-8}					
Slc7a2	4.42×10^{-9}					
Scd1		4.68×10^{-5}, 0.48	0.011, 0.4	0.001, 0.51		
Elovl6		0.003, 0.36	0.04, 0.32	0.013, 0.41		
Alanine	0.003	0.04, -0.42	0.043, -0.42		0.001, -0.62	0.003, 0.57
Methionine	0.013	0.045, -0.38	0.045, -0.36		0.01, -0.51	0.02, 0.47
Valine	0.019	0.036, -0.43	0.046, -0.39		0.015, -0.49	0.011, 0.51
Serine	0.023	0.045, -0.41	0.032, -0.44		0.01, -0.49	0.014, 0.49
Arginine	< 0.001	3.82×10^{-5}, -0.74	1.13×10^{-4}, -0.71	0.002, -0.59	5×10^{-4}, - 0.66	3.9×10^{-6}, 0.79
Lysine	1.95×10^{-5}	0.005, -0.55	0.031, -0.44	0.017, -0.48	2.8×10^{-5}, -0.77	3.9×10^{-5}, 0.73
Leucine	< 0.001	0.001, -0.63	0.006, -0.54	0.02, -0.46	4×10^{-4}, -0.67	3.5×10^{-5}, 0.74
Histidine	7.68×10^{-5}	7.5×10^{-4}, -0.64	0.002, -0.6	0.006, -0.54	8.36×10^{-5}, -0.72	2.1×10^{-5}, 0.75
Bilirubin		0.041, -0.42	0.037, -0.42			
S1P		0.023, -0.46	0.016, -0.49			
Linoleic acid		0.034, -0.43				

Table S11. P and correlation coefficients of correlation analysis between expression levels of genes and metabolites.

Metabolites	*Got1*	*Slc43a1*	*G6pc*	*Pck1*
Alanine	0.006<br>-0.55	3×10^{-4}<br>-0.67	0.04<br>-0.43	0.0008<br>-0.64
Arginine	2.84×10^{-5}<br>-0.74	2.14×10^{-5}<br>-0.75	0.0007<br>-0.64	0.003<br>-0.58
Histidine	3.92×10^{-5}<br>-0.73	6.55×10^{-6}<br>0.78	4.18×10^{-6}<br>-0.79	8.54×10^{-5}<br>-0.71
Leucine	3.82×10^{-5}<br>-0.73	3.86×10^{-6}<br>-0.79	0.0006<br>-0.65	0.001<br>-0.62
Lysine	9.28×10^{-5}<br>-0.71	3.69×10^{-5}<br>-0.74	0.002<br>-0.61	0.0003<br>-0.67
Methionine	0.011<br>-0.51	0.002<br>-0.61		
Serine	0.001<br>-0.61	7.77×10^{-5}<br>-0.72	0.03<br>-0.45	0.004<br>-0.57
Valine	0.01<br>-0.5	0.003<br>-0.58		

Table S12. 60%fat and different protein content diet composition table.

Product #	5 kcal% Protein		10 kcal % Protein		15 kcal % Protein		20 kcal % Protein		25 kcal % Protein		30 kcal % Protein	
	**gm%**	**kcal%**	**gm%**	**kcal%**	**gm%**	**kcal%**	**kcal%**	**kcal%**	**gm%**	**kcal%**	**gm%**	**kcal%**
**Protein**	**7**	**5**	**13**	**10**	**20**	**15**	**26**	**20**	**33**	**25**	**39**	**30**
Carbohydrate	46	35	39	30	33	25	26	20	20	15	13	10
**Fat**	**35**	**60**	**35**	**60**	**35**	**60**	**35**	**60**	**35**	**60**	**35**	**60**
Total		100		100		100		100		100		100
kcal/gm	5.2		5.2		5.2		5.2		5.2		5.1	
**Ingredient**	**gm**	**kcal**	**gm**	**kcal**	**gm**	**kcal**	**gm**	**kcal**	**gm**	**kcal**	**gm**	**kcal**
Casein	50	200	100	400	150	600	200	800	250	1000	300	1200
L-Cystine	0.75	3	1.5	6	2.25	9	3	12	3.75	15	4.5	18
Corn Starch	145	580	114.3	457	68.6	274	17.8	71	0	0	0	0
Maltodextrin 10	150	600	130	520	125	500	125	500	92	368	41.2	165
Sucrose	51	204	51	204	51	204	51	204	51	204	51	204
Cellulose, BW200	50	0	50	0	50	0	50	0	50	0	50	0
Cocoa Butter	75	675	75	675	75	675	75	675	75	675	75	675
Coconut Oil	5	45	5	45	5	45	5	45	5	45	5	45
Menhaden Oil	5	45	5	45	5	45	5	45	5	45	5	45
Palm Oil	165	1485	165	1485	165	1485	165	1485	165	1485	165	1485
Safflower Oil	20	180	20	180	20	180	20	180	20	180	20	180
Mineral Mix, S10026	10	0	10	0	10	0	10	0	10	0	10	0
DiCalcium Phosphate	13	0	13	0	13	0	13	0	13	0	13	0
Calcium Carbonate	5.5	0	5.5	0	5.5	0	5.5	0	5.5	0	5.5	0
Potassium Citrate, 1 H2O	16.5	0	16.5	0	16.5	0	16.5	0	16.5	0	16.5	0
Vitamin Mix, V10001	10	40	10	40	10	40	10	40	10	40	10	40
Choline Bitartrate	2	0	2	0	2	0	2	0	2	0	2	0
FD&C Yellow Dye #5	0	0	0	0	0	0	0	0	0	0	0	0
FD&C Red Dye #40	0.0125	0	0.05	0	0.075	0	0.125	0	0.175	0	0.2	0
FD&C Blue Dye #1	0	0	0	0	0	0	0	0	0	0	0	0
**Total**	**773.763**	**4057**	**773.85**	**4057**	**773.925**	**4057**	**773.925**	**4057**	**773.925**	**4057**	**773.925**	**4057**

Mice treated with these 6 diets were analyzed for liver RNA seq and serum metabolomics.

Table S13. 20%fat and different protein content diet composition table.

Product #	5 kcal% Protein		10 kcal % Protein		15 kcal % Protein		20 kcal % Protein		25 kcal % Protein		30 kcal % Protein	
	**gm%**	**kcal%**	**gm%**	**kcal%**	**gm%**	**kcal%**	**kcal%**	**kcal%**	**gm%**	**kcal%**	**gm%**	**kcal%**
**Protein**	**5**	**5**	**10**	**10**	**15**	**15**	**20**	**20**	**25**	**25**	**30**	**30**
Carbohydrate	76	75	71	70	66	65	61	60	56	55	51	50
**Fat**	**9**	**20**	**9**	**20**	**9**	**20**	**9**	**20**	**9**	**20**	**9**	**20**
Total		100		100		100		100		100		100
kcal/gm	4.1		4.1		4.1		4.1		4.1		4.1	
**Ingredient**	**gm**	**kcal**	**gm**	**kcal**	**gm**	**kcal**	**gm**	**kcal**	**gm**	**kcal**	**gm**	**kcal**
Casein	50	200	100	400	150	600	200	800	250	1000	300	1200
L-Cystine	0.75	3	1.5	6	2.25	9	3	12	3.75	15	4.5	18
Corn Starch	600	2400	549.3	2197	498.5	1994	447.7	1791	397	1588	346.2	1385
Maltodextrin 10	100	400	100	400	100	400	100	400	100	400	100	400
Sucrose	51	204	51	204	51	204	51	204	51	204	51	204
Cellulose, BW200	50	0	50	0	50	0	50	0	50	0	50	0
Cocoa Butter	25	225	25	225	25	225	25	225	25	225	25	225
Coconut Oil	1.7	15	1.7	15	1.7	15	1.7	15	1.7	15	1.7	15
Menhaden Oil	1.7	15	1.7	15	1.7	15	1.7	15	1.7	15	1.7	15
Palm Oil	55	495	55	495	55	495	55	495	55	495	55	495
Safflower Oil	6.6	59	6.6	59	6.6	59	6.6	59	6.6	59	6.6	59
Mineral Mix, S10026	10	0	10	0	10	0	10	0	10	0	10	0
DiCalcium Phosphate	13	0	13	0	13	0	13	0	13	0	13	0
Calcium Carbonate	5.5	0	5.5	0	5.5	0	5.5	0	5.5	0	5.5	0
Potassium Citrate, 1 H2O	16.5	0	16.5	0	16.5	0	16.5	0	16.5	0	16.5	0
Vitamin Mix, V10001	10	40	10	40	10	40	10	40	10	40	10	40
Choline Bitartrate	2	0	2	0	2	0	2	0	2	0	2	0
FD&C Yellow Dye #5	0.0063	0	0.025	0	0.0375	0	0.0625	0	0.0875	0	0.1	0
FD&C Red Dye #40	0.0063	0	0.025	0	0.0375	0	0.0625	0	0.0875	0	0.1	0
FD&C Blue Dye #1	0	0	0	0	0	0	0	0	0	0	0	0
**Total**	**998.76**	**4057**	**998.85**	**4057**	**998.83**	**4057**	**998.83**	**4057**	**998.83**	**4057**	**998.9**	**4057**

Mice treated with these 6 diets were analyzed for liver RNA seq and serum metabolomics.

Table S14. 10% protein and different fat content diet composition table.

Product #	10 kcal% Fat		30 kcal% Fat		40 kcal% Fat		50 kcal% Fat		70 kcal% Fat		80 kcal% Fat	
	**gm%**	**kcal%**	**gm%**	**kcal%**	**gm%**	**kcal%**	**kcal%**	**kcal%**	**gm%**	**kcal%**	**gm%**	**kcal%**
**Protein**	**10**	**10**	**11**	**10**	**11**	**10**	**12**	**10**	**14**	**10**	**15**	**10**
Carbohydrate	77	80	65	60	57	50	49	40	28	20	15	10
**Fat**	**4**	**10**	**14**	**30**	**20**	**40**	**27**	**50**	**44**	**70**	**54**	**80**
Total		100		100		100		100		100		100
kcal/gm	3.8		4.3		4.6		4.9		5.7		6.1	
**Ingredient**	**gm**	**kcal**	**gm**	**kcal**	**gm**	**kcal**	**gm**	**kcal**	**gm**	**kcal**	**gm**	**kcal**
Casein	100	400	100	400	100	400	100	400	100	400	100	400
L-Cystine	1.5	6	1.5	6	1.5	6	1.5	6	1.5	6	1.5	6
Corn Starch	650.6	2602	448	1792	347	1388	245.5	982	43.2	173	0	0
Maltodextrin 10	100	400	100	400	100	400	100	400	100	400	41.4	166
Sucrose	51	204	51	204	51	204	51	204	51	204	51	204
Cellulose, BW200	50	0	50	0	50	0	50	0	50	0	50	0
Cocoa Butter	12.5	113	37.5	338	50	450	62.5	563	87.5	788	100	900
Coconut Oil	0.85	8	2.5	23	3.3	30	4.2	38	5.8	52	6.7	60
Menhaden Oil	0.85	8	2.5	23	3.3	30	4.2	38	5.8	52	6.7	60
Palm Oil	27.5	248	82.5	743	110	990	137.5	1238	192.5	1733	220	1980
Safflower Oil	3.3	30	10	90	13.3	120	16.6	149	23.3	210	26.7	240
Mineral Mix, S10026	10	0	10	0	10	0	10	0	10	0	10	0
DiCalcium Phosphate	13	0	13	0	13	0	13	0	13	0	13	0
Calcium Carbonate	5.5	0	5.5	0	5.5	0	5.5	0	5.5	0	5.5	0
Potassium Citrate, 1 H2O	16.5	0	16.5	0	16.5	0	16.5	0	16.5	0	16.5	0
Vitamin Mix, V10001	10	40	10	40	10	40	10	40	10	40	10	40
Choline Bitartrate	2	0	2	0	2	0	2	0	2	0	2	0
FD&C Yellow Dye #5	0.0063	0	0.025	0	0.0375	0	0.0625	0	0.0875	0	0.125	0
FD&C Red Dye #40	0	0	0	0	0	0	0	0	0	0	0	0
FD&C Blue Dye #1	0.0063	0	0.025	0	0.0375	0	0.0625	0	0.0875	0	0.125	0
**Total**	**1055**	**4057**	**942.55**	**4057**	**886.48**	**4057**	**830.13**	**4057**	**717.78**	**4057**	**661.25**	**4057**

Mice treated with these 6 diets were analyzed for serum metabolomics.

Table S15. 25% protein and different fat content diet composition table.

Product #	8.3 kcal% Fat		25 kcal% Fat		33.3 kcal% Fat		41.7 kcal% Fat		58.3 kcal% Fat		66.6 kcal% Fat	
	**gm%**	**kcal%**	**gm%**	**kcal%**	**gm%**	**kcal%**	**kcal%**	**kcal%**	**gm%**	**kcal%**	**gm%**	**kcal%**
**Protein**	**24**	**25**	**26**	**25**	**27**	**25**	**29**	**25**	**32**	**25**	**34**	**25**
Carbohydrate	64	67	52	50	46	42	39	33	22	17	12	8
**Fat**	**4**	**8.3**	**12**	**25**	**16**	**33.3**	**21**	**41.7**	**34**	**58.3**	**41**	**66.6**
Total		100		100		100		100		100		100
kcal/gm	3.8		4.2		4.4		4.6		5.2		6.1	
**Ingredient**	**gm**	**kcal**	**gm**	**kcal**	**gm**	**kcal**	**gm**	**kcal**	**gm**	**kcal**	**gm**	**kcal**
Casein	250	1000	250	1000	250	1000	250	1000	250	1000	250	1000
L-Cystine	3.75	15	3.75	15	3.75	15	3.75	15	3.75	15	3.75	15
Corn Starch	515.5	2062	346.5	1386	262	1048	176.5	706	8	32	0	0
Maltodextrin 10	100	400	100	400	100	400	100	400	100	400	24	96
Sucrose	51	204	51	204	51	204	51	204	51	204	51	204
Cellulose, BW200	50	0	50	0	50	0	50	0	50	0	50	0
Cocoa Butter	10.37	93	31.25	281	41.67	375	52.22	470	73.03	657	83.39	751
Coconut Oil	0.69	6	2.08	19	2.78	25	3.48	31	4.87	44	5.56	50
Menhaden Oil	0.69	6	2.08	19	2.78	25	3.48	31	4.87	44	5.56	50
Palm Oil	22.8	205	68.75	619	91.67	825	114.89	1034	160.66	1446	183.45	1651
Safflower Oil	2.77	25	8.33	75	11.1	100	13.92	125	19.47	175	22.24	200
Mineral Mix, S10026	10	0	10	0	10	0	10	0	10	0	10	0
DiCalcium Phosphate	13	0	13	0	13	0	13	0	13	0	13	0
Calcium Carbonate	5.5	0	5.5	0	5.5	0	5.5	0	5.5	0	5.5	0
Potassium Citrate, 1 H2O	16.5	0	16.5	0	16.5	0	16.5	0	16.5	0	16.5	0
Vitamin Mix, V10001	10	40	10	40	10	40	10	40	10	40	10	40
Choline Bitartrate	2	0	2	0	2	0	2	0	2	0	2	0
FD&C Yellow Dye #5	0	0	0	0	0	0	0	0	0	0	0	0
FD&C Red Dye #40	0	0	0	0	0	0	0	0	0	0	0	0
FD&C Blue Dye #1	0.0125	0	0.05	0	0.075	0	0.125	0	0.175	0	0.225	0
**Total**	**1064.6**	**4057**	**970.79**	**4057**	**923.83**	**4057**	**876.37**	**4057**	**782.83**	**4057**	**736.18**	**4057**

Mice treated with these 6 diets were analyzed for serum metabolomics.
